# Gait dynamics and brain function abnormalities in Parkinson’s disease with freezing of gait: a clinical study using resting-state fMRI and wearable devices

**DOI:** 10.3389/fnins.2025.1560333

**Published:** 2025-07-03

**Authors:** Xiaohong Li, Mengdi Hou, Yan Qu, Yuan Huo, Shuting Liu, Minghui Ma, Zhanhua Liang

**Affiliations:** ^1^Department of Neurology, First Affiliated Hospital of Dalian Medical University, Dalian, Liaoning Province, China; ^2^Department of Radiology, Changshu Hospital Affiliated to Nantong University, Jiangsu, China; ^3^Department of Neurology, Affiliated Dalian Municipal Friendship Hospital of Dalian Medical University, Dalian, Liaoning Province, China

**Keywords:** parkinson’s disease, freezing of gait, resting-state fMRI, gait dynamics, amplitude of low-frequency fluctuations

## Abstract

**Introduction:**

Parkinson’s disease (PD)-associated freezing of gait (FoG) (PDFoG) refers to episodes where patients feel the urge to move but experience temporary immobility or markedly shortened steps. This leads to frequent falls and, eventually, the loss of walking ability, severely affecting patient quality of life and life expectancy. Despite its clinical importance, the neural mechanisms underlying PDFoG remain unclear.

**Methods:**

This study sought to characterize abnormal neural activity in PDFoG by assessing regional brain activity using ALFF, fALFF, PerAF, and wavelet-ALFF across three frequency bands (conventional, slow-5, and slow-4). PDFoG patients were compared to PD patients without FoG (PDnFoG) and healthy controls. Clinical evaluations included standard assessment scales, such as the FOG-Q and MDS-UPDRS III, alongside a wearable sensor-based gait assessment system.

**Results:**

We found that PD patients with FoG experienced more extensive changes in regional brain activity than those without FoG, primarily affecting cortical regions and the cerebellum. Conversely, PDnFoG patients primarily showed reduced activity in the basal ganglia.

**Conclusion:**

These findings emphasize the need to further explore the roles of the cerebral cortex and cerebellum in PDFoG pathophysiology.

## 1 Introduction

Freezing of gait (FoG) is a common symptom of Parkinson’s disease (PD), characterized by brief episodes of halted or markedly reduced forward stepping despite the intention to walk. Patients often describe this as a sensation of their feet being “glued to the floor” (Nutt et al., 2011). FoG becomes more prevalent as PD progresses, severely impacting mobility and quality of life while raising the risk of falls (Kwok et al., 2022). FoG can be triggered or worsened by specific situations, such as turning, navigating narrow passages, or increased motor (e.g., obstacle crossing), cognitive (e.g., dual-tasking), or limbic (e.g., anxiety) load, suggestive of a complex underlying pathophysiology involving the dysfunction of multiple brain structures (Droby et al., 2021; Gilat et al., 2018a).

Several non-mutually exclusive theories have been proposed to explain the pathogenesis of PD-associated FoG (PDFoG). One theory posits that automaticity of movement is disrupted due to impairment of the basal ganglia, which plays a critical role in controlling automatic motor actions (Nutt et al., 2011). Skilled movements, such as walking, are automatic and require little attention. In PDFoG patients, stepping often relies on a conscious effort or external cues, potentially attributable to the loss of this automaticity. This may explain why FoG frequently occurs when other motor or cognitive tasks are being performed, given that competing inputs from motor, cognitive, and limbic cortical areas may cause the output nuclei of the basal ganglia to fire synchronously (i.e., become overloaded), leading to the over-inhibition of the brainstem locomotor system, and thereby triggering freezing (Nutt et al., 2011). Focusing on goal-directed action to reset basal ganglia output or using external cues (e.g., lines on the floor) may help “break the freeze” (Nutt et al., 2011). Additional hypotheses include perceptual malfunction and frontal executive dysfunction (i.e., difficulties with set-shifting, attention, problem-solving, and response inhibition) (Nutt et al., 2011). However, the mechanism underlying FoG remains incompletely understood.

Magnetic resonance imaging (MRI) studies have reported that functional and structural integrity is compromised in PDFoG patients (Bharti et al., 2019; Canu et al., 2015; Droby et al., 2021; Tessitore et al., 2012; Wang et al., 2016). Functional imaging offers an advantage over structural imaging in studying PDFoG as functional deficits may precede structural alterations in both white and gray matter in early-stage PD patients, enabling opportunities for early intervention (Droby et al., 2022; Morris et al., 2023). Resting-state functional MRI (rs-fMRI) is a modality for studying brain function at rest (i.e., in the absence of tasks or external stimuli). It provides insight into the brain’s intrinsic functional networks and has been applied in neurodegenerative disorders such as PD and dementia (Peraza et al., 2014; Wolters et al., 2019). To date, most research employing rs-fMRI in PDFoG has focused on functional connectivity and network parameters to investigate the neural underpinnings of FoG. These studies reported that functional connectivity is disrupted within resting-state cortical networks, as well as between subcortical structures and cortical regions (Bharti et al., 2019; Canu et al., 2015; Droby et al., 2021; Fling et al., 2014; Gilat et al., 2018b; Maidan et al., 2019; Tessitore et al., 2012; Wang et al., 2016). In contrast, relatively few rs-fMRI-based studies have explored regional activity changes related to PDFoG. However, given the extensive disruptions in functional connectivity reported in these works, examining local activity patterns in PDFoG is crucial for identifying the aberrant regional activity potentially underlying these observed disruptions. Prior research (Hu et al., 2020; Mi et al., 2017) evaluated local spontaneous neural activity in PDFoG patients using amplitude of low-frequency fluctuation (ALFF). Compared to PD patients without FOG (PDnFoG) and healthy controls (HCs), PDFoG patients showed either increased or decreased ALFF values in the cerebellum, thalamus, striatum, cingulate cortex, and the frontal, parietal, and temporal lobes (Mi et al., 2017).

In the present study, expanding on prior research on ALFF in PDFoG, we evaluated regional spontaneous activity by quantifying low-frequency (0.01–0.08 Hz) oscillatory strength using a suite of metrics. These included ALFF, fractional ALFF (fALFF), percent amplitude of fluctuation (PerAF), and wavelet transform-based ALFF (wavelet-ALFF). While ALFF is sensitive to the intensity of the low-frequency fluctuations characteristic of the brain’s resting-state activity, it is susceptible to physiological noise (Zang et al., 2007). fALFF, the ratio of the power in the low-frequency range (0.01–0.08 Hz) to the entire frequency spectrum (0–0.25 Hz), can suppress physiological noise in cisterns while enhancing signals from cortical regions (Zou et al., 2008). PerAF, which measures percent signal change per volume relative to the mean time series intensity, has demonstrated greater short- and long-term test-retest reliability than ALFF and fALFF (Jia et al., 2020). Wavelet-ALFF, employing wavelet transforms for signal decomposition, may offer higher sensitivity in detecting differences between groups and conditions than the Fourier-based ALFF approach (Luo et al., 2020).

Mi et al. (2017) assessed ALFF only within the conventional frequency band (0.01–0.08 Hz). However, different brain regions are known to oscillate at distinct frequency ranges (Buzsáki and Draguhn, 2004), with gray matter-related oscillations primarily occurring in the slow-4 and slow-5 bands (Zuo et al., 2010). Building on this, Hu et al. (2020) measured ALFF in the conventional band (0.01–0.08 Hz) as well as in its slow-5 (0.01–0.027 Hz) and slow-4 (0.027–0.073 Hz) sub-bands. They reported ALFF differences among PDFoG patients, PDnFoG patients, and HCs in the temporal cortex (conventional, slow-4), frontal cortex (slow-4), and putamen (slow-5). These findings highlight the importance of frequency-dependent analyses in elucidating the neural underpinnings of PDFoG, particularly in the slow-5 and slow-4 bands.

In this study, we measured ALFF, fALFF, PerAF, and wavelet-ALFF in PD patients with FoG, PD patients without FoG, and healthy individuals across three frequency bands, namely, conventional, slow-5, and slow-4. This approach enabled the capture of metric- and frequency-specific regional oscillatory changes in PDFoG. Because PDFoG disrupts multiple neural circuits and likely impacts numerous brain regions, our comprehensive evaluation of regional spontaneous activity may reflect these complex mechanisms. Consistent with previous research, we anticipated finding widespread brain abnormalities in PDFoG patients. Our findings advance the understanding of regional dysfunctions in PDFoG and inform the development of targeted neuromodulatory interventions.

## 2 Materials and methods

### 2.1 Participants

The cohort in this study included idiopathic PD patients recruited from the Department of Neurology at the First Affiliated Hospital of Dalian Medical University and the Department of Neurology at Dalian Municipal Friendship Hospital and HCs recruited from the health examination center. This study was conducted in accordance with the latest version of the Declaration of Helsinki and was approved by the Ethics Committees of the First Affiliated Hospital of Dalian Medical University (Ethical approval number PJ-KS-KY-2021-259(X)) and Dalian Municipal Friendship Hospital (Ethical approval number YY-LL-2021-048). All participants provided informed consent before being enrolled in the study and underwent scanning at the Dalian Municipal Friendship Hospital.

The participants were divided into three groups—PDFoG, PDnFoG, and HC. The inclusion criteria for the PDFoG group were (1) aged over 18 years and meeting the 2015 International Parkinson and Movement Disorder Society Clinical Diagnostic Criteria for Parkinson’s Disease (Postuma et al., 2015); (2) Hoehn and Yahr (H-Y) stage between 2 and 3 (on medication), able to walk without assistance or walking aids; (3) on regular medication prior to enrolment, with stable symptoms, and no unpredictable “on-off phenomena” or uncontrolled dyskinesia; and (4) a Freezing of Gait Questionnaire (FOG-Q) score ≥ 1 in item 3, with episodes of FoG confirmed by a neurologist during initiation, turning, or passing through narrow passages. The inclusion criteria for the PDnFoG group were identical to those of the PDFoG group, except that participants did not exhibit FoG.

The exclusion criteria were as follows: (1) secondary Parkinsonism or Parkinson’s plus syndrome; (2) Mini-Mental State Examination (MMSE) score (Folstein et al., 1975) < 21; (3) history of deep brain stimulation (DBS) or other brain surgery; (4) psychiatric or other neurological disorder; (5) coexisting conditions affecting gait, such as musculoskeletal diseases; (6) left-handedness; and (7) inability to cooperate with clinical scales, gait assessments, or MRI.

PD patients were scanned after being off medication for more than 12 h. Medication was withheld to eliminate potential drug effects on the MRI images. Both MRI and sensor-based gait assessments were conducted in the morning while patients were in the L-dopa OFF state. After resuming their medication, patients underwent clinical scale evaluations in the ON state. Medication was restarted beforehand because the evaluation was lengthy, and, without it, patients would likely feel unwell and struggle to complete the assessments.

The clinical scales included MDS-UPDRS III, H-Y staging (Goetz et al., 2008), the FOG-Q, Hamilton Anxiety Scale (HAMA) (14-item version) (Shear et al., 2001), Hamilton Depression Scale (HAMD) (24-item version) (Hamilton, 1960), MMSE, Montreal Cognitive Assessment (MoCA) (Nasreddine et al., 2005), Pittsburgh Sleep Quality Index (PSQI), Epworth Sleepiness Scale (ESS), and the 39-item Parkinson’s Disease Questionnaire (PDQ-39). HCs were required to complete the HAMA, HAMD, MMSE, and MoCA assessments. All assessments were performed by two neurologists who were qualified to administer the scales.

### 2.2 Gait assessment using wearable sensors

#### 2.2.1 Gait kinematic data

The wearable motion and gait quantitative evaluation system, RuiPing (MATRIX, MA11, GYENNO SCIENCE Co., Ltd., Shenzhen, China), developed by Shenzhen Zhenluo Science & Technology Co., was used to analyze typical gait disorders in the PDFoG and PDnFoG groups. The system consists of 10 wireless high-precision sensors and provides real-time output of 200 quantitative parameters (Lin et al., 2023).

In this study, patients performed the following three motion paradigms (Supplementary Figure 1): (1) Timed Up and Go (TUG): Starting seated, patients walked 5 meters, turned, and returned to sit (He et al., 2024; Zhang W. et al., 2024); (2) Narrow Path Walking (NPW): similar to TUG, except that the patient walked through a 0.6-meter-wide path on both the forward and return trajectories (Almeida and Lebold, 2010; Cowie et al., 2012); (3) Turning: Patients stood in a 0.6-meter square, turned 360 degrees counter-clockwise, paused for 10 s, and then turned 360 degrees clockwise (Mancini et al., 2017; Snijders et al., 2012).

Fourteen parameters were analyzed, including TUG gait speed (m/s), step length (cm), cadence (steps/min), stride length (cm), gait cycle (s), and sit-to-stand duration (s); NPW gait speed (m/s) and step length (cm); Turning mean step count (steps) and mean angular velocity (°/s); TUG stride velocity asymmetry (%) and absolute deviation (m/s); and NPW stride velocity asymmetry (%) and absolute deviation (m/s). Among these parameters, higher values for TUG gait cycle (s), TUG sit-to-stand duration (s), and Turning mean step count (steps) indicate more severe gait impairment. Higher values for TUG and NPW stride velocity asymmetry (%) and absolute deviation (m/s) reflect greater asymmetry in lower limb activity. For the remaining seven parameters, lower values indicate more severe gait disturbances.

#### 2.2.2 Freeze index (FI)

FI was collected via sensors mounted around both ankles while patients performed the TUG and NPW paradigms. FI output from the Turning paradigm is currently unavailable due to technical limitations, precluding the device from providing FI values for this condition (Ren et al., 2022). This index, developed by Moore et al. (2008)<mergref>Moore et al. (2013)</mergref>, leverages the characteristic high-frequency leg trembling observed during FoG to enable objective and automated detection of FoG episodes. Although the FI generally performs well in distinguishing FoG from normal gait events (i.e., FoG-provoking situations without freezing), it may be less reliable during voluntary stopping or in non-trembling types of FoG, such as shuffling or akinesia (Cockx et al., 2023). In our cohort, the vast majority of patients exhibited the trembling type of FoG. The FI at time *t* is calculated using a sliding time window centered at *t*, based on the ratio of the squared area under the curve (AUC) of the power spectrum in the “freeze” band (3–8 Hz) to that in the “locomotion” band (0.5–3 Hz) (Moore et al., 2008), as defined by the following formula:


$$F\,I=\frac{{\left(A\,U\,C\,o\,f\,p\,o\,w\,e\,r\,s\,p\,e\,c\,t\,r\,u\,m\,i\,n\,f\,r\,e\,e\,z\,e\,b\,a\,n\,d\right)}^{2}}{{\left(A\,U\,C\,o\,f\,p\,o\,w\,e\,r\,s\,p\,e\,c\,t\,r\,u\,m\,i\,n\,l\,o\,c\,o\,m\,o\,t\,i\,o\,n\,b\,a\,n\,d\right)}^{2}}$$

In the present study, a 2-s rectangular sliding window was applied with 0.5-s steps, and data were sampled at 100 Hz. The resulting FI values were then normalized by multiplying by 100 and taking the natural logarithm, as proposed by Moore et al. (2008). FI values were computed for three directions of leg movement: vertical (up–down), left–right, and front–back. We focused on FI values derived from vertical leg acceleration in the NPW condition. This was because passing through narrow passages (NPW paradigm) is more likely to elicit FoG in patients with PD than walking straight in open spaces (TUG paradigm) (Nutt et al., 2011). Additionally, vertical-axis FI has been shown to provide the most discriminative and consistently reliable signal for FI-based FoG detection (Pham et al., 2017). Each movement axis produced two FI metrics: the maximum FI value (FI_max_) and the average FI value (FI_avg_). FI_max_ captures the highest FI observed within a given period and is particularly useful for identifying freezing episodes, whereas FI_avg_ represents the mean FI over time and is commonly used to gauge the overall severity of freezing behavior. Prior studies have used either or both indices (Cockx et al., 2023; Hoyos et al., 2019; Moore et al., 2008). In the present study, both FI_max_ and FI_avg_ were analyzed to provide a comprehensive evaluation of freezing severity. FI_max_ was defined as the higher value of the two legs, while FI_avg_ was calculated as the mean of both legs. Higher FI values indicate more severe freezing.

### 2.3 rs-fMRI data acquisition

Each participant underwent 3D structural imaging and rs-fMRI scanning. All scans were performed using a 3.0T MAGNETOM Skyra DE (SIEMENS Healthineers) machine. During scanning, participants lay supine on the scanner table with their heads secured by a foam pad to minimize motion. They were instructed to relax, keep their eyes closed, and avoid falling asleep. The fMRI data were acquired with the following parameters: repetition time (TR) = 2,000 ms, echo time (TE) = 30 ms, 35 slices, thickness = 4 mm, gap = 0.6 mm, matrix = 64 × 64, flip angle = 90°, field of view (FOV) = 240 × 240 mm, voxel size = 3.75 × 3.75 × 4.0 mmł, 240 volumes; scan time = 8 min. High-resolution T1-weighted images were acquired with the following parameters: TR = 2,300 ms, TE = 2.32 ms, flip angle = 8°, thickness = 0.9 mm, gap = 0 mm, FOV = 240 × 240 mm, voxel size = 1 × 1 × 1 mmł; scan time = 5 min 15 s.

### 2.4 rs-fMRI data processing

#### 2.4.1 Preprocessing

Resting-state fMRI data underwent pre- and post-processing using the Resting-State fMRI Data Analysis Toolkit Plus (RESTplus) v.1.27^1^ (Jia et al., 2019) in MATLAB R2017b (The MathWorks Inc., Natick, MA, United States). The following preprocessing steps were applied: conversion of DICOM images to the NIFTI format; deletion of the first 10 volumes (leaving 230 volumes); slice time correction; realignment to correct for slight head motion, with participants whose head motion exceeded 3 mm or 3° being excluded; spatial normalization to the Montreal Neurological Institute (MNI) space using the new segment method, with resampling to 3-mm isotropic voxels; smoothing with a 6-mm full-width at half-maximum (FWHM) Gaussian kernel; linear detrending; and nuisance regression. In the regression model, Friston-24 head motion parameters (Friston et al., 1996), cerebrospinal fluid and white matter signals, and the global mean signal (Power et al., 2017) of each participant were included as covariates. For PerAF, the “add mean back” option was selected during nuisance regression, and band-pass filtering in three frequency bands of interest (conventional: 0.01–0.08 Hz, slow-5: 0.01–0.027 Hz, and slow-4: 0.027–0.073 Hz) was applied during preprocessing. For ALFF, fALFF, and wavelet-ALFF, the same band-pass filtering was applied during post-processing.

#### 2.4.2 Post-processing

RESTplus uses a GUI-based pipeline that requires minimal user input. Metric calculation required user input only for specifying the frequency bands of interest. For all other computations, the default parameters of the toolbox were applied. The preprocessed time series of each voxel was band-pass filtered and transformed into the frequency domain *via* fast Fourier transform (FFT) to obtain the power spectrum. The square root of the power at each frequency was then computed and averaged across the frequency range to obtain the ALFF value for each voxel (Zang et al., 2007). The voxel-wise fALFF value was calculated as the sum of amplitudes within the frequency range of interest divided by the sum of amplitudes across the entire frequency range (0–0.25 Hz) (Zou et al., 2008).

The PerAF for each voxel was derived using the following formula:


$$P\,e\,r\,A\,F=\frac{1}{n}\,\sum_{i=1}^{n}\left|\frac{X_{i}-\mu }{\mu }\right|\times 100\%$$

$$\mu =\frac{1}{n}\,\sum_{i=1}^{n}X_{i}$$

where *Xi* represents the signal intensity of the *i^th^* volume, μ is the mean signal intensity of the time series, and *n* is the total number of volumes in the time series (Jia et al., 2020).

Wavelet-ALFF was calculated using the following formulas:


$$C\,W\,T\,\left(k,s\right)=\frac{1}{\sqrt{s}}\cdot \int_{-\infty }^{+\infty }x\,\left(t\right)\cdot \psi^{*}\,\left(\frac{t-k}{s}\right)\,dt$$

$$W\,a\,v\,e\,l\,e\,t-A\,L\,F\,F=\frac{1}{m}\,\sum_{i=1}^{n}\left|C\,W\,T_{i,j}\right|,j=s_{1}\,\cdots \,s_{m}$$

where *x*(*t*)denotes the time series of a voxel, ψ_*k*,*s*_(*t*) denotes the mother wavelet function (db2 in this case), *s* denotes the wavelet scale, *k* denotes the localized time index, and * denotes the complex conjugate. |*CWT*_*i*,*j*_| represents the absolute value of the wavelet coefficient at volume *i* for a given frequency bin *j*, *n* is the total number of wavelet coefficients at a given frequency bin, while *m* denotes the total number of frequency bins within a given frequency band (Luo et al., 2020). The preprocessed time series were decomposed into the time-frequency domain through continuous wavelet transform (CWT), wherein the time series was convolved with scaled and translated versions of the Daubechies 2 (db2) wavelet function. CWT coefficients were obtained for all volumes at each of the 64 frequency bins within the 0–0.25 Hz range. Wavelet-ALFF was then computed as the average of these coefficients over all volumes within a given frequency band (Luo et al., 2020).

For standardization, the ALFF, fALFF, PerAF and wavelet-ALFF of each voxel were divided by their respective global mean values within a default whole-brain mask.

### 2.5 Statistical analysis

Demographic and clinical data were analyzed in GraphPad Prism 9 (GraphPad Software, Boston, MA, United States). The statistical tests used are specified in the caption of Table 1. The tests were selected based on the data type, normality, and variance of the data distribution. All tests were two-tailed, and a *p*-value of < 0.05 was considered significant.

**TABLE 1:** Demographic and clinical characteristics of the PDFoG patients, PDnFoG patients and healthy controls (HC).

Characteristics	PDFoG (*n* = 39)	PDnFoG (*n* = 40)	HC (*n* = 44)	Test statistics	*p* values
**Demographics**					
Sex (female, %)	20 (51.28%)	17 (42.5%)	23 (52.27%)	^1^χ(2) = 0.94	*p* = .624
Age (mean ± SD, years)	70.97 ± 8.82	68.58 ± 8.78	69.43 ± 4.23	^2^*W* = 0.76	*p* = .470
Education (mean ± SD, years)	12.18 ± 3.19	10.73 ± 3.90	11.30 ± 2.59	^2^*W* = 1.78	*p* = .176
**Clinical characteristics**					
Duration of illness (mean ± SD, years)	8.51 ± 4.20	3.11 ± 2.51	NA	^3^*t* = 6.91, *df* = 61.74	*p* < .001
MoCA (mean ± SD)	21.44 ± 4.64	21.23 ± 3.70	22.64 ± 2.69	^2^*W* = 2.36	*p* = .101
MMSE (mean ± SD)	26.56 ± 2.98	27.05 ± 2.76	26.20 ± 2.41	^4^*H* = 3.82	*p* = .148
HAMA (mean ± SD)	20.36 ± 10.88	17.68 ± 9.49	8.64 ± 4.17	^2^PDFoG vs HC: *t* = 6.33, *df*	*p* < .001
				= 47.81	
				^2^PDnFoG vs HC: *t* = 5.56, *df*	*p* < .001
				= 52.41	
HAMD (mean ± SD)	15.49 ± 8.11	12 ± 6.54	4.5 ± 3.17	^2^PDFoG vs HC: *t* = 7.94, *df*	*p* < .001
				= 48.23	
				^2^PDnFoG vs HC: *t* = 6.58, *df*	*p* < .001
				= 55.19	
PSQI (mean ± SD)	9.97 ± 4.84	8.98 ± 4.75	7.41 ± 3.76	^4^PDFoG vs HC: *z* = 2.51	*p* = .036
H-Y stage (median, 1st & 3rd quartiles)	3 (2.5, 3)	2.25 (2, 3)	NA	^5^*U* = 393	*p* < .001
LEDD (mean ± SD)	686.18 ± 244.82	375.98 ± 268.44	NA	^5^*U* = 262.5	*p* < .001
FOG-Q (mean ± SD)	18.23 ± 4.16	0.75 ± 1.21	NA	^3^*t* = 25.19, *df* = 44.27	*p* < .001
UPDRS III (mean ± SD)	40 ± 13.65	28.4 ± 12.40	NA	^6^*t* = 3.96, *df* = 77	*p* < .001
ESS (mean ± SD)	8.18 ± 6.60	4.6 ± 5.30	NA	^5^*U* = 524	*p* = .011
PDQ-39 (mean ± SD)	53.74 ± 28.95	23.58 ± 16.30	NA	^3^*t* = 5.69, *df* = 59.56	*p* < .001
TUG: Step length (cm)	36.01 ± 15.02	47.90 ± 12.59	NA	^6^*t* = 3.82, *df* = 77	*p* < .001
TUG: Gait speed (m/s)	0.65 ± 0.29	1.84 ± 6.27	NA	^3^*t* = 1.20, *df* = 39.17	*p* = .236
TUG: Cadence (steps/min)	110.44 ± 11.19	103.23 ± 23.69	NA	^3^*t* = 1.74, *df* = 55.88	*p* = .088
TUG: Stride length (cm)	72.61 ± 29.83	95.23 ± 25.17	NA	^6^*t* = 3.65, *df* = 77	*p* < .001
TUG: Gait cycle (s)	1.12 ± 0.13	1.15 ± 0.14	NA	^6^*t* = 1.02, *df* = 77	*p* = .309
TUG: Sit-to-stand duration (s)	3.72 ± 4.46	1.86 ± 0.68	NA	^3^*t* = 2.58, *df* = 39.70	*p* = .014
NPW: Step length (cm)	35.70 ± 15.10	47.42 ± 12.95	NA	^6^*t* = 3.71, *df* = 77	*p* < .001
NPW: Gait speed (m/s)	0.65 ± 0.28	0.85 ± 0.25	NA	^6^*t* = 3.33, *df* = 77	*p* = .001
Turning: Mean angular velocity (degree/s)	47.96 ± 27.18	73.39 ± 26.24	NA	^5^*U* = 355	*p* < .001
Turning: Mean step count (step)	42.42 ± 21.52	19.94 ± 9.10	NA	^3^*t* = 6.02, *df* = 50.90	*p* < .001
TUG: Stride velocity asymmetry (%)	9.01 ± 6.25	6.72 ± 2.36	NA	^3^*t* = 2.14, *df* = 48.41	*p* = .037
TUG: Stride velocity absolute deviation (m/s)	0.05 ± 0.02	0.06 ± 0.02	NA	^5^*U* = 685.5	*p* = .351
NPW: Stride velocity asymmetry (%)	10.19 ± 5.91	7.63 ± 2.72	NA	^3^*t* = 2.46, *df* = 53.06	*p* = .017
NPW: Stride velocity absolute deviation (m/s)	0.06 ± 0.03	0.06 ± 0.03	NA	^5^*U* = 676	*p* = .306
NPW: FI_max_ (vertical)	1.44 ± 0.43	1.05 ± 0.19	NA	^3^*t* = 5.16, *df* = 51.49	*p* < .001**#**
NPW: FI_avg_ (vertical)	0.48 ± 0.27	0.37 ± 0.30	NA	^5^*U* = 545	*p = .*021∧

1. Chi-square test; 2. Welch’s ANOVA & Dunnett’s T3 multiple comparisons test; 3. Welch’s *t*-test; 4. Kruskal-Wallis test & Dunn’s multiple comparisons test; 5. Mann-Whitney test; 6. Two-sample *t*-test. *df* = degrees of freedom. PDFoG = Parkinson’s Disease with freezing of gait, PDnFoG = Parkinson’s Disease without freezing of gait; MoCA = Montreal Cognitive Assessment; MMSE = Mini-Mental State Examination; HAMA = Hamilton Anxiety Rating Scale; HAMD = Hamilton Depression Rating Scale; PSQI = Pittsburgh Sleep Quality Index; H-Y stage = Hoehn and Yahr Staging Scale; LEDD = Levodopa Equivalent Daily Dose; FOG-Q = the Freezing of Gait Questionnaire; UPDRS III = Unified Parkinson’s Disease Rating Scale Part III (Motor Examination); ESS = Epworth Sleepiness Scale; PDQ-39 = Parkinson’s Disease Questionnaire-39; TUG = the Timed Up and Go test; NPW = the narrow path walking condition; Turning = the assessment of a person’s ability to turn; FI_max_ (vertical) = maximum freeze index derived from vertical linear acceleration of the shank; FI_avg_ (vertical) = average freeze index derived from vertical linear acceleration of the shank. **#** and ∧ Data were log-transformed prior to statistical testing due to severe deviation from normality and unequal variances; ∧ After excluding an outlier from the PDnFoG group, *t* = 2.69, *df* = 76, *p* = .009 (two-sample *t*-test).

The standardized ALFF, fALFF, PerAF, and wavelet-ALFF of the three groups of participants—PDFoG, PDnFoG, and HCs—were compared pairwise using independent samples *t*-tests in RESTplus v.1.27. Duration of illness was included as a covariate. Corrections for multiple comparisons were performed using Gaussian Random Field (GRF) theory (voxel-level *p* < 0.05, cluster-level *p* < 0.05, two-tailed). The 116-anatomical region Automated Anatomical Labeling (AAL) gray matter mask was employed for statistical comparisons and correction for multiple comparisons.

Next, discriminatory FoG-nFoG regional activity amplitudes were correlated with gait parameters and FI values (FI_max_ and FI_avg_) in the PDFoG group. The standardized ALFF, fALFF, PerAF, and wavelet-ALFF values in the three frequency bands (conventional, slow-5, and slow-4) were extracted from brain areas showing significant differences between the two patient groups (i.e., PDFoG *vs*. PDnFoG, detailed in Tables 2–5), using the MNI coordinate of the peak *t*-value as the center and a 6-mm radius. For all four metrics and three frequency bands, each significant brain area was correlated with the gait parameters and FI values one by one. In GraphPad Prism 9, Spearman’s rank-order correlation was used because some gait parameters and regional activity amplitudes did not satisfy the assumption of normality under the Shapiro-Wilk test. The Benjamini-Hochberg method (Benjamini and Hochberg, 1995) was then applied to control the false discovery rate (*q* = 0.05).

**TABLE 2:** Differences in ALFF between PDFoG patients, PDnFoG patients and healthy controls (HC).

						MNI coordinate (mm)		
Metric	Frequency bands	^1^Comparisons	Brain regions (aal)	Cluster size	Peak *t(p)* values	x	y	z
ALFF	Conventional: 0.01–0.08 Hz	PDFoG vs PDnFoG	Rectus_R	181	–3.7589 (< .001)	3	24	–15
			Postcentral_R	458	4.2148 (< .001)	42	–33	54
		PDFoG vs HC	Fusiform_R	673	3.8422 (< .001)	42	–54	–24
			Postcentral_L	881	4.9452 (< .001)	–57	–9	36
			Postcentral_R	677	4.3761 (< .001)	45	–27	45
		PDnFoG vs HC	Frontal_Inf_Orb_L	546	4.3371 (< .001)	–48	30	–12
			Putamen_R	299	–4.3451 (< .001)	21	15	0
	Slow-5: 0.01–0.027 Hz	PDFoG vs PDnFoG	Frontal_Med_Orb_R	378	–4.3122 (< .001)	3	45	–12
			Postcentral_L	168	3.9582 (< .001)	-57	–9	36
			Postcentral_R	371	3.8848 (< .001)	54	–6	30
		PDFoG vs HC	Cerebelum_6_L	555	4.705 (< .001)	–12	–60	–15
			Calcarine_R	163	4.483 (< .001)	18	–51	9
			Parietal_Sup_L	774	4.5535 (< .001)	–30	–48	60
			Postcentral_R	747	4.4606 (< .001)	39	–30	39
		PDnFoG vs HC	Temporal_Mid_L	297	4.0786 (< .001)	–57	3	–30
			Pallidum_R	455	–4.9664 (< .001)	18	6	–3
	Slow–4: 0.027 to 0.073 Hz	PDFoG vs PDnFoG	Postcentral_R	395	4.5396 (< .001)	42	–33	54
		PDFoG vs HC	Fusiform_R	258	4.0655 (< .001)	42	–54	–24
			Vermis_3	264	3.6317 (< .001)	6	–36	–6
			Postcentral_L	750	4.717 (< .001)	–57	–9	36
			Precentral_R	579	4.0573 (< .001)	48	–21	57
		PDnFoG vs HC	Fusiform_L	309	4.1553 (< .001)	-36	–42	–21
			Putamen_R	162	–3.6471 (< .001)	21	15	6

1. The latter group was subtracted from the former. PDFoG = patients with Parkinson’s Disease and freezing of gait; PDnFoG = patients with Parkinson’s Disease and no freezing of gait. Degrees of freedom (*df*) for PDFoG vs PDnFoG is 76; *df* for PDFoG vs HC is 80; *df* for PDnFoG vs HC is 81.

**TABLE 3:** Differences in fALFF between PDFoG patients, PDnFoG patients and healthy controls (HC).

						MNI coordinate (mm)		
Metric	Frequency bands	^1^Comparisons	Brain regions (aal)	Cluster size	Peak *t(p)* values	x	y	z
fALFF	Conventional: 0.01-0.08 Hz	PDFoG vs PDnFoG	Frontal_Med_Orb_R	225	–4.1114 (< .001)	6	45	–12
			Frontal_Inf_Oper_R	222	4.3741 (< .001)	57	12	27
		PDFoG vs HC	Fusiform_L	190	4.5071 (< .001)	–36	–15	–30
			Olfactory_R	171	–3.9455 (< .001)	3	27	0
			Calcarine_L	689	5.0076 (< .001)	–18	–63	12
			Postcentral_R	479	4.8442 (< .001)	33	–27	45
			Postcentral_L	278	4.0549 (< .001)	–48	–24	54
		PDnFoG vs HC	Paracentral_Lobule_L	159	4.4066 (< .001)	–9	–33	69
	Slow-5: 0.01-0.027 Hz	PDFoG vs PDnFoG	Lingual_L	160	3.8873 (< .001)	–12	–84	–12
			Frontal_Med_Orb_R	455	–5.1185 (< .001)	3	42	–12
			Frontal_Inf_Oper_R	241	4.2118 (< .001)	54	6	21
			Precentral_L	151	4.8329 (< .001)	–57	0	36
		PDFoG vs HC	Cerebelum_4_5_R	193	3.9809 (< .001)	21	–48	–21
			Olfactory_R	243	–4.5706 (< .001)	3	27	0
			Calcarine_L	827	4.2296 (< .001)	0	–81	–12
			Occipital_Mid_L	246	3.9319 (< .001)	–27	–87	21
			Frontal_Sup_Medial_L	175	–3.5495 (< .001)	–9	18	42
			Postcentral_R	300	4.4201 (< .001)	33	–30	42
			Parietal_Sup_L	173	3.5661 (< .001)	–36	–45	63
		PDnFoG vs HC	Caudate_R	163	–4.2168 (< .001)	6	12	–3
	Slow–4: 0.027 to 0.073 Hz	PDFoG vs PDnFoG	Temporal_Mid_L	122	4.5916 (< .001)	–48	–27	–15
			Cerebelum_4_5_L	225	–4.5351 (< .001)	–6	–60	–9
			Frontal_Inf_Oper_R	125	4.4323 (< .001)	54	12	30
		PDFoG vs HC	Calcarine_L	538	4.6922 (< .001)	–21	–63	12
			Angular_R	222	–3.6053 (< .001)	57	–60	33
			Postcentral_L	208	4.189 (< .001)	–48	–24	60
			Postcentral_R	224	4.01 (< .001)	51	–18	51
		PDnFoG vs HC	Cerebelum_Crus1_L	173	–4.1018 (< .001)	–54	–60	–33

1. The latter group was subtracted from the former. PDFoG = patients with Parkinson’s Disease and freezing of gait; PDnFoG = patients with Parkinson’s Disease and no freezing of gait. Degrees of freedom (*df*) for PDFoG vs PDnFoG is 76; *df* for PDFoG vs HC is 80; *df* for PDnFoG vs HC is 81.

**TABLE 4:** Differences in PerAF between PDFoG patients, PDnFoG patients and healthy controls (HC).

						MNI coordinate (mm)		
Metric	Frequency bands	^1^Comparisons	Brain regions (aal)	Cluster size	Peak *t(p)* values	x	y	z
PerAF	Conventional: 0.01–0.08 Hz	PDFoG vs PDnFoG	Cerebelum_6_R	733	4.6419 (< .001)	12	–63	–15
			Postcentral_R	869	4.1361 (< .001)	66	0	24
			Postcentral_L	489	4.4966 (< .001)	–57	–9	36
		PDFoG vs HC	Calcarine_R	977	4.7713 (< .001)	18	–51	9
			Postcentral_R	1046	5.7974 (< .001)	66	–9	33
			Postcentral_L	848	5.341 (< .001)	–60	–9	36
		PDnFoG vs HC	Cerebelum_Crus2_R	306	3.6737 (< .001)	18	–81	–45
			Frontal_Med_Orb_L	465	–4.1322 (< .001)	–6	45	–12
			Putamen_R	201	–3.6693 (< .001)	21	15	6
	Slow–5: 0.01 to 0.027 Hz	PDFoG vs PDnFoG	Cerebelum_6_R	541	5.0185 (< .001)	12	–63	–15
			Rectus_R	259	–4.2469 (< .001)	6	27	–15
			Postcentral_R	730	4.1317 (< .001)	66	–3	24
			Postcentral_L	374	4.1425 (< .001)	–57	–9	36
		PDFoG vs HC	Cerebelum_6_L	793	5.4817 (< .001)	–12	–60	–15
			Frontal_Med_Orb_L	193	–3.8555 (< .001)	–3	45	–9
			Postcentral_L	784	5.1363 (< .001)	–60	–6	36
			Precentral_R	1047	5.7228 (< .001)	57	3	48
		PDnFoG vs HC	Fusiform_R	179	3.7143 (< .001)	33	–75	–18
			Caudate_R	683	–4.6669 (< .001)	6	12	–3
	Slow–4: 0.027 to 0.073 Hz	PDFoG vs PDnFoG	Vermis_8	220	3.4133 (0.001)	6	–63	–27
			Cerebelum_Crus1_R	203	4.0713 (< .001)	45	-48	–27
			Postcentral_R	814	4.2619 (< .001)	42	–30	54
			Postcentral_L	447	4.3065 (< .001)	–57	–9	36
		PDFoG vs HC	Cerebelum_4_5_R	1008	4.3169 (< .001)	9	–45	–12
			Postcentral_R	978	5.7839 (< .001)	66	–9	33
			Postcentral_L	829	4.9194 (< .001)	–60	–6	36
		PDnFoG vs HC	Frontal_Med_Orb_L	265	–3.856 (< .001)	–6	45	–9

1. The latter group was subtracted from the former. PDFoG = patients with Parkinson’s Disease and freezing of gait; PDnFoG = patients with Parkinson’s Disease and no freezing of gait. Degrees of freedom (*df*) for PDFoG vs PDnFoG is 76; *df* for PDFoG vs HC is 80; *df* for PDnFoG vs HC is 81.

**TABLE 5:** Differences in wavelet-ALFF between PDFoG patients, PDnFoG patients and healthy controls (HC).

						MNI coordinate (mm)		
Metric	Frequency bands	^1^Comparisons	Brain regions (aal)	Cluster size	Peak *t(p)* values	x	y	z
Wavelet-ALFF	Conventional: 0.01-0.08 Hz	PDFoG vs PDnFoG	Postcentral_L	270	4.0991 (< .001)	–57	–9	36
			Postcentral_R	379	4.2666 (< .001)	42	–33	54
		PDFoG vs HC	Cerebelum_6_R	256	4.0072 (< .001)	39	–54	–24
			Cuneus_R	377	3.8923 (< .001)	9	–81	30
			Postcentral_L	851	5.002 (< .001)	–57	–9	36
			Postcentral_R	607	4.118 (< .001)	39	–33	54
		PDnFoG vs HC	Frontal_Inf_Orb_L	611	4.3085 (< .001)	–48	30	–12
			Caudate_R	403	–4.5726 (< .001)	9	3	9
	Slow–5: 0.01 to 0.027 Hz	PDFoG vs PDnFoG	Frontal_Med_Orb_R	329	–4.0554 (< .001)	3	45	–12
			Postcentral_R	458	3.753 (< .001)	66	–3	24
			Postcentral_L	172	3.922 (< .001)	–57	–9	36
		PDFoG vs HC	Cerebelum_6_L	545	4.4105 (< .001)	–12	–60	–15
			Frontal_Inf_Orb_R	169	4.1906 (< .001)	45	39	–9
			Calcarine_R	201	4.6913 (< .001)	18	–51	9
			Postcentral_R	741	4.4108 (< .001)	45	–27	45
			Postcentral_L	846	4.8623 (< .001)	–57	–9	36
		PDnFoG vs HC	Temporal_Inf_L	380	3.9271 (< .001)	–57	–3	–27
			Pallidum_R	267	–4.4676 (< .001)	18	9	0
			Putamen_L	238	–4.9331 (< .001)	–21	9	0
	Slow-4: 0.027-0.073 Hz	PDFoG vs PDnFoG	Postcentral_R	346	4.4465 (< .001)	42	–33	54
		PDFoG vs HC	Cerebelum_6_R	249	4.0324 (< .001)	39	–54	–24
			Cuneus_R	362	3.8847 (< .001)	9	–81	30
			Postcentral_L	824	4.8207 (< .001)	–57	–9	36
			Postcentral_R	554	4.1646 (< .001)	48	–21	57
		PDnFoG vs HC	Cerebelum_4_5_L	494	3.9271 (< .001)	–30	–33	–30
			Caudate_R	320	–4.6385 (< .001)	9	3	9

1. The latter group was subtracted from the former. PDFoG = patients with Parkinson’s Disease and freezing of gait; PDnFoG = patients with Parkinson’s Disease and no freezing of gait. Degrees of freedom (*df*) for PDFoG vs PDnFoG is 76; *df* for PDFoG vs HC is 80; *df* for PDnFoG vs HC is 81.

### 2.6 Validation analysis

Given that head motion and smoothing kernel size can influence rs-fMRI results (Liu et al., 2017; Maknojia et al., 2019; Sacchet and Knutson, 2013), we assessed the robustness of the findings by testing different kernel sizes and implementing additional motion control. As 4, 6, and 8 mm are commonly used smoothing kernels, we repeated the analysis using 4 and 8 mm kernels for comparison. Head motion had already been addressed during preprocessing through realignment and regression of the Friston-24 motion parameters (Friston et al., 1996). To further control for motion, mean framewise displacement was included as a covariate in the statistical analyses (Power et al., 2012).

## 3 Results

### 3.1 Demographic and clinical characteristics

**FIGURE 1:**
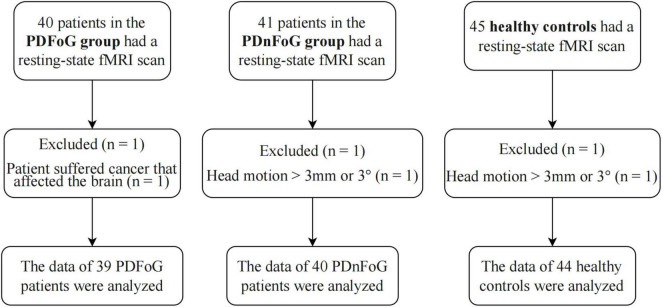
Flowchart of the participant exclusion and inclusion process.

Eighty-one patients with PD and forty-five HCs received an MRI scan. Of the patients, 40 were diagnosed as PDFoG, while the remaining 41 were identified as PDnFoG. Following the exclusion of 1 PDFoG patient due to brain-related cancer, along with 1 PDnFoG patient and 1 HC due to excessive head movement, a total of 39 PDFoG patients, 40 PDnFoG patients, and 44 HCs were included in the analysis (Figure 1).

The demographic and clinical profiles of the PDFoG patients, PDnFoG patients, and HCs are displayed in Table 1. There were no significant differences in age, sex, or years of education among the groups. On average, PDnFoG patients had a significantly shorter duration of illness (*t* = 6.91, *df* = 61.74, *p* < 0.001) and took a lower daily medication dose (LEDD: *U* = 262.5, *p* < 0.001) than PDFoG patients. They also scored lower on motor symptoms (H-Y staging: *U* = 393, *p* < 0.001; FOG-Q: *t* = 25.19, *df* = 44.27, *p* < 0.001; UPDRS III: *t* = 3.96, *df* = 77, *p* < 0.001), the impact of PD on quality of life (PDQ-39: *t* = 5.69, *df* = 59.56, *p* < 0.001), and daytime sleepiness (ESS: *U* = 524, *p* = 0.011). No significant differences in anxiety (HAMA), depression (HAMD), or night-time sleep quality (PSQI) were found between the two patient groups, although their scores on these scales were significantly higher than those of the HCs. The only exception was that PDnFoG patients did not significantly differ from HCs in PSQI ratings. No significant difference in cognitive status, as measured by MoCA and MMSE, was detected among the three groups.

As expected, the two patient groups displayed significantly different gait behaviors (Table 1). In the TUG test, PDFoG patients exhibited a shorter step length (cm; *t* = 3.82, *df* = 77, *p* < 0.001) and stride length (cm; *t* = 3.65, *df* = 77, *p* < 0.001), a longer sit-to-stand duration (s; *t* = 2.58, *df* = 39.70, *p* = 0.014), and greater stride velocity asymmetry (%; *t* = 2.14, *df* = 48.41, *p* = 0.037); however, there was no significant difference in gait speed (m/s), cadence (steps/min), gait cycle (s), or stride velocity absolute deviation (m/s) between the two groups. In the narrow path walking assessment, PDFoG patients were characterized by a shorter step length (cm; *t* = 3.71, *df* = 77, *p* < 0.001), slower gait speed (m/s; *t* = 3.33, *df* = 77, *p* = 0.001), greater stride velocity asymmetry (%; *t* = 2.46, *df* = 53.06, *p* = 0.017), and similar stride velocity absolute deviation (m/s; *U* = 676, *p* = 0.306). In the assessment of Turning, PDFoG patients demonstrated a lower mean angular velocity (°/s; *U* = 355, *p* < 0.001) and a higher mean step count (steps; *t* = 6.02, *df* = 50.90, *p* < 0.001). Additionally, PDFoG patients showed higher FI_max_ (*t* = 5.16, *df* = 51.49, *p* < 0.001) and FI_avg_ (*U* = 545, *p* = 0.021) values during the narrow path walking condition.

### 3.2 rs-fMRI: ALFF, fALFF, PerAF, and wavelet-ALFF

#### 3.2.1 ALFF

**FIGURE 2:**
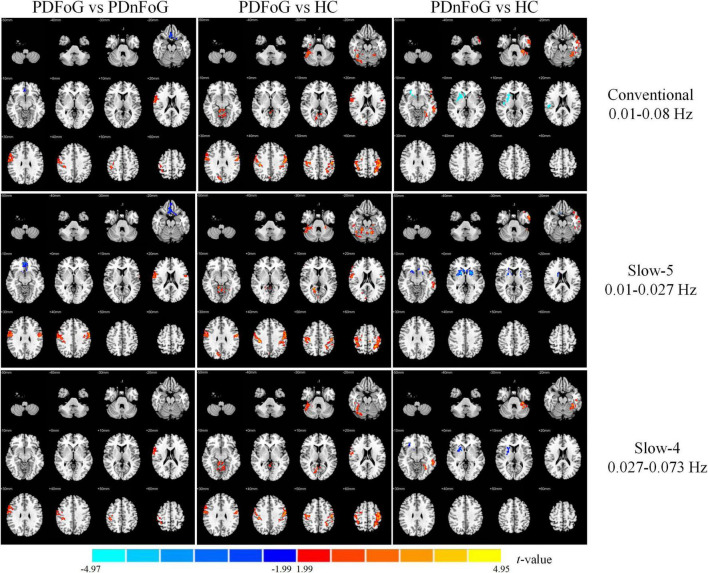
Group differences in ALFF across three frequency bands for PDFoG vs PDnFoG, PDFoG vs HC, and PDnFoG vs HC. The color bar represents *t*-values, with warm colors indicating increased ALFF and cool colors indicating decreased ALFF in the former group of each comparison (e.g., the PDFoG group in the comparison PDFoG vs PDnFoG). PDFoG = patients with Parkinson’s Disease and freezing of gait; PDnFoG = patients with Parkinson’s Disease and no freezing of gait; HC = healthy controls.

Table 2 and Figure 2 present the between-group differences in ALFF across the three frequency bands. Compared to PDnFoG patients, PDFoG patients showed higher ALFF in the right postcentral gyrus across all three frequency bands, and the left postcentral gyrus in the slow-5 band. Conversely, ALFF was decreased in the right rectus gyrus and right superior frontal gyrus (medial orbital) in the conventional and slow-5 bands, respectively.

Compared to HCs, PDFoG patients demonstrated increased ALFF in the bilateral postcentral gyri and the right fusiform gyrus in the conventional and slow-4 bands; the right postcentral gyrus, right calcarine fissure and surrounding cortex, left cerebellar lobule VI, and left superior parietal gyrus in the slow-5 band; and vermis lobule III in the slow-4 band.

In contrast, PDnFoG patients showed reduced ALFF in the right putamen in the conventional and slow-5 bands, and the right pallidum in the slow-4 band compared to HCs. Increased ALFF was seen in the left inferior frontal gyrus (pars orbitalis), left fusiform gyrus, and left middle temporal gyrus in the conventional, slow-5, and slow-4 bands, respectively.

#### 3.2.2 fALFF

**FIGURE 3:**
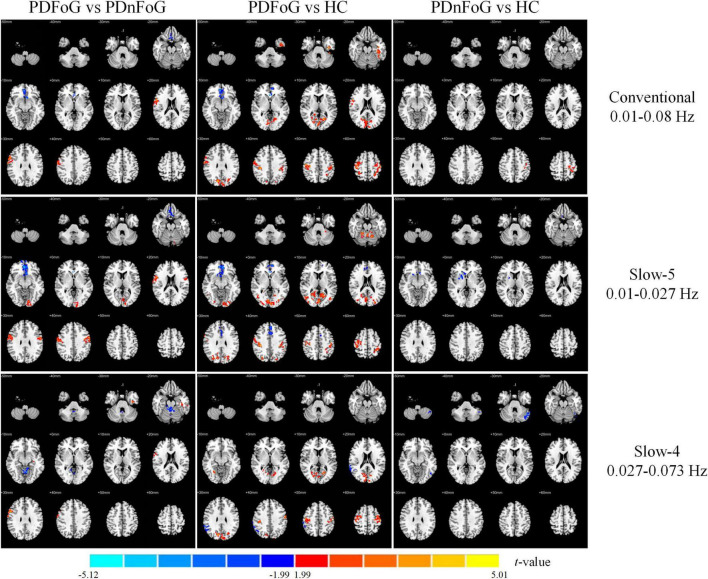
Group differences in fALFF across three frequency bands for PDFoG vs PDnFoG, PDFoG vs HC, and PDnFoG vs HC. The color bar represents *t*-values, with warm colors indicating increased fALFF and cool colors indicating decreased fALFF in the first group of each comparison (e.g., the PDFoG group in the comparison PDFoG vs PDnFoG). PDFoG = patients with Parkinson’s Disease and freezing of gait; PDnFoG = patients with Parkinson’s Disease and no freezing of gait; HC = healthy controls.

The between-group differences in fALFF across the three frequency bands are summarized in Table 3 and Figure 3. Compared to PD patients without FoG, those with FoG displayed higher fALFF in the right inferior frontal gyrus (opercular part) across all three bands; the left lingual gyrus and left precentral gyrus in the slow-5 band; and the left middle temporal gyrus in the slow-4 band. Meanwhile, fALFF was reduced in the right superior frontal gyrus (medial orbital) in the conventional and slow-5 bands, and the left cerebellar lobules IV and V in the slow-4 band.

Compared to HCs, PDFoG patients had increased fALFF in the left calcarine fissure and surrounding cortex across all three bands, the bilateral postcentral gyri in the conventional and slow-4 bands, and the left fusiform gyrus in the conventional band. The right cerebellar lobules IV and V, right postcentral gyrus, left middle occipital gyrus, and left superior parietal gyrus showed increases in the slow-5 band, whereas the opposite was detected in the right olfactory cortex in the conventional and slow-5 bands, the left medial superior frontal gyrus in the slow-5 band, and the right angular gyrus in the slow-4 band.

Furthermore, PDnFoG patients demonstrated higher fALFF values in the left paracentral lobule in the conventional band, and lower fALFF values in the right caudate and left cerebellar crus I in the slow-5 and slow-4 bands, respectively, relative to HCs.

#### 3.2.3 PerAF

**FIGURE 4:**
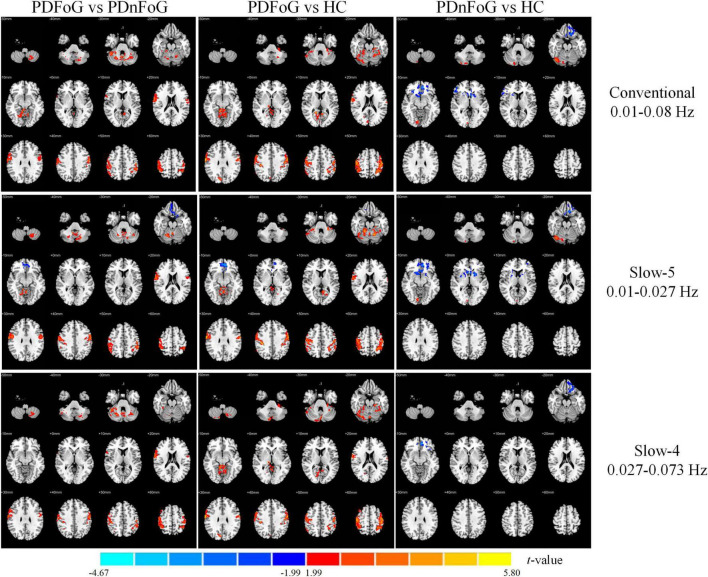
Group differences in PerAF across three frequency bands for PDFoG vs PDnFoG, PDFoG vs HC, and PDnFoG vs HC. The color bar represents *t*-values, with warm colors indicating increased PerAF and cool colors indicating decreased PerAF in the first group of each comparison (e.g., the PDFoG group in the comparison PDFoG vs PDnFoG). PDFoG = patients with Parkinson’s Disease and freezing of gait; PDnFoG = patients with Parkinson’s Disease and no freezing of gait; HC = healthy controls.

Table 4 and Figure 4 detail the between-group differences in PerAF across the three frequency bands. Compared to PDnFoG patients, PDFoG patients showed higher PerAF in the bilateral postcentral gyri across all three bands, the right cerebella lobule VI in the conventional and slow-5 bands, and Vermis Lobule VIII and the right cerebellar crus I in the slow-4 band. A decrease in PerAF was observed in the right rectus gyrus in the slow-5 band.

Relative to HCs, PDFoG patients exhibited elevated PerAF in the bilateral postcentral gyri (conventional and slow-4 bands); the right calcarine fissure and surrounding cortex (conventional band), the left postcentral gyrus, the right precentral gyrus, and the left cerebellar lobule VI in the slow-5 band; and the right cerebellar lobules IV and V in the slow-4 band. A reduction in PerAF was noted in the left superior frontal gyrus (medial orbital) in the slow-5 band.

Finally, compared to HCs, PDnFoG patients displayed reduced PerAF in the left superior frontal gyrus (medial orbital) in the conventional and slow-4 bands, the right putamen in the conventional band, and the right caudate in the slow-5 band. However, PerAF was found to be increased in the right cerebellar Crus II in the conventional band and the right fusiform gyrus in the slow-5 band.

#### 3.2.4 Wavelet-ALFF

**FIGURE 5:**
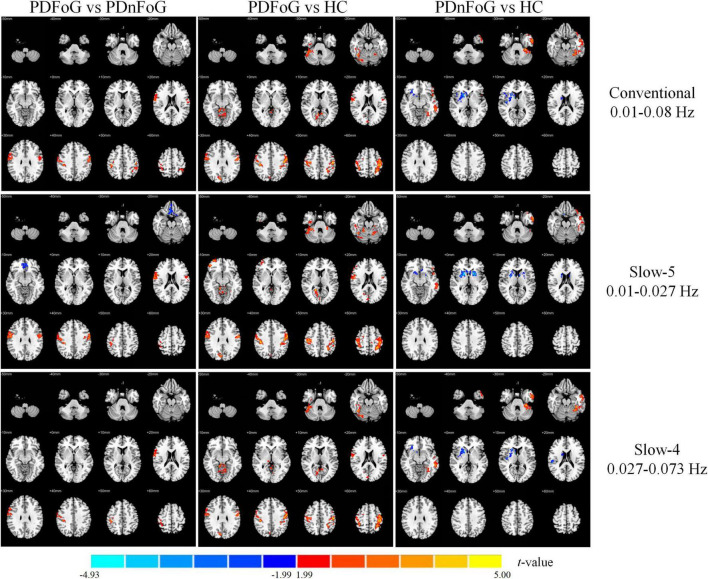
Group differences in wavelet-ALFF across three frequency bands for PDFoG vs PDnFoG, PDFoG vs HC, and PDnFoG vs HC. The color bar represents *t*-values, with warm colors indicating increased wavelet-ALFF and cool colors indicating decreased wavelet-ALFF in the first group of each comparison (e.g., the PDFoG group in the comparison PDFoG vs PDnFoG). PDFoG = patients with Parkinson’s Disease and freezing of gait; PDnFoG = patients with Parkinson’s Disease and no freezing of gait; HC = healthy controls.

Table 5 and Figure 5 present between-group differences in wavelet-ALFF across the three frequency bands. Compared to PDnFoG patients, PDFoG patients showed higher wavelet-ALFF in the bilateral postcentral gyri in the conventional and slow-5 bands, and the right postcentral gyrus in the slow-4 band. In contrast, PD patients with FoG exhibited lower wavelet-ALFF in the right superior frontal gyrus (medial orbital) in the slow-5 band than those without FoG.

Meanwhile, relative to PDnFoG patients, PDFoG patients showed increased wavelet-ALFF in the bilateral postcentral gyri across all three bands; the right cuneus and right cerebellar lobule VI in the conventional and slow-4 bands; and the left cerebellar lobule VI, right inferior frontal gyrus (pars orbitalis), and right calcarine fissure and surrounding cortex in the slow-5 band.

Additionally, PDnFoG patients showed decreased wavelet-ALFF in the right caudate in the conventional and slow-4 bands, and the left putamen and right pallidum in the slow-5 band compared to HCs. Conversely, compared with HCs, PDFoG patients exhibited higher wavelet-ALFF in the left inferior frontal gyrus (pars orbitalis) in the conventional band, the left inferior temporal gyrus in the slow-5 band, and the left cerebellar lobules IV and V in the slow-4 band.

### 3.3 Correlational analysis

No significant correlation was found between FoG-related regional activity amplitudes and sensor-based gait parameters, FI_max_ or FI_avg_ in PDFoG patients.

### 3.4 Validation analysis

The spatial patterns of results obtained with 4- and 8-mm smoothing kernels, as well as those obtained after accounting for mean framewise displacement, resembled our original findings. Supplementary Figures 2–4 present the spatial distributions of the results of the validation analysis.

## 4 Discussion

In this study, we examined regional spontaneous activity in both PDFoG and PDnFoG patients using four rs-fMRI metrics—ALFF, fALFF, PerAF, and wavelet-ALFF. These measures were analyzed across three frequency bands, namely, the conventional band (0.01–0.08 Hz) and its two sub-bands, slow-5 (0.01–0.027 Hz) and slow-4 (0.027–0.073 Hz). We also performed a correlation analysis between regional activity, extracted from brain areas showing significant differences between FoG and nFoG patients, and gait parameters and FI values in the PDFoG group.

Overall, there was a greater number of abnormal brain regions in PDFoG patients than in PDnFoG patients relative to HCs, indicating that FoG is associated with more widespread abnormalities in local brain activity. Among these regions, the bilateral postcentral gyri showed the most pronounced differences across metrics and frequency bands in PDFoG patients; in contrast, no significant differences were observed in these brain regions in PDnFoG patients. Specifically, compared to PD patients without FoG, those with FoG exhibited higher ALFF, PerAF, and wavelet-ALFF across all three frequency bands in the right postcentral gyrus. In the left postcentral gyrus, PDFoG patients showed higher PerAF and wavelet-ALFF across all three bands, along with higher ALFF and fALFF in the slow-5 band. Compared to HCs, PDFoG patients demonstrated higher ALFF, fALFF, and wavelet-ALFF across all three bands, as well as increased PerAF in the conventional and slow-4 bands in the right postcentral gyrus. In the left postcentral gyrus, they showed higher PerAF and wavelet-ALFF across all three bands and increased ALFF and fALFF in the conventional and slow-4 bands.

The primary somatosensory cortex (S1), located in the postcentral gyrus, plays a crucial role in generating skilled movement. It translates and integrates sensory signals from multiple modalities (e.g., vision, hearing, touch, proprioception), both before and during movement, into a real-time “body-status” report—indicating where the body is and how it is interacting with the world—that the motor system uses to generate precise motor commands for smooth, coordinated movements (Borich et al., 2015). S1 closely interacts with other motor and sensory regions, and manipulating its activity directly impacts visual-motor integration and fine motor control (Borich et al., 2015; Brodie et al., 2014; Vidoni et al., 2010). Previous rs-fMRI studies have shown that connectivity between S1 and the parietal operculum, corresponding to the secondary somatosensory area (S2), is lower in PDFoG patients than in PDnFoG patients (Lenka et al., 2016). Similarly, connectivity between S1 and other areas of the sensorimotor network, such as the precentral gyrus, supplementary motor area, and superior frontal gyrus, was reported to be lower in PDFoG patients than in HCs (Canu et al., 2015). These findings suggest that functional integration between the postcentral gyrus and other sensorimotor areas is weakened in PDFoG. In the current study, we observed that spontaneous activity in the bilateral postcentral gyri was higher in PDFoG patients than in PDnFoG patients and HCs. This finding may be interpreted in the context of the perceptual malfunction theory of FoG (Nutt et al., 2011), which posits that freezing—often occurring when patients attempt to walk through doorways or narrow passages—cannot be explained by a simple visual-perceptual deficit, as patients can correctly judge doorway width while seated. Rather, such freezing may be attributable to an exaggerated response to action-relevant visual information, leading to impaired online planning of locomotor adaptation. Our results may support this theory, in that increased spontaneous activity in the bilateral postcentral gyri may predispose these regions to abnormally amplified responses when facing environmental changes. This, in turn, may disrupt visual-motor integration, that is, the ability to form an appropriate motor plan based on visual input. The bilateral postcentral gyri may thus serve as potential targets for the application of non-invasive neurostimulation techniques, such as repetitive transcranial magnetic stimulation (rTMS) or transcranial direct current stimulation (tDCS), in the treatment of PDFoG.

Some brain regions demonstrated frequency-specific activity differences, with significant differences seen in either the slow-5 or slow-4 frequency sub-bands, but not both. This inconsistency across sub-bands may be explained by each region having a preferred oscillatory frequency through which it connects with the rest of the brain (Gong and Zuo, 2023). For instance, in the right cerebellar lobule VI, PDFoG patients showed higher activity in the slow-5 and slow-4 bands than PDnFoG patients and HCs, respectively. In the left cerebellar lobule VI, PDFoG patients exhibited increased activity in the slow-5 band compared to HCs. Cerebellar lobule VI has been identified as part of the intrinsic (functionally connected at rest) sensorimotor network and, among cerebellar regions, shows the strongest correlations with several sensorimotor cortical areas, including the motor, premotor, somatosensory, visual (middle temporal), and auditory (superior temporal) cortices (Habas et al., 2009; O’Reilly et al., 2010). This suggests that cerebellar lobule VI serves as a core region supporting sensorimotor integration. In our study, the peak differences in the slow-5 band between the PDFoG and the other two groups were located near the vermis, within bilateral cerebellar lobule VI (MNI coordinates [12, −63, −15] and [−12, −60, −15]). These peaks likely correspond to the paramedian clusters of cerebellar lobule VI, identified by Habas et al. (2009) as forming part of the sensorimotor network. In contrast, the peak difference in the slow-4 band between PDFoG patients and HCs in the right cerebellar lobule VI (39, −54, −24) was more lateral, corresponding to the lateral clusters associated with the salience detection network (Habas et al., 2009). This functional delineation of cerebellar lobule VI supports the findings of Dobromyslin et al. (2012), who reported the existence of functional connectivity between bilateral cerebellar lobule VI and sensorimotor cortical areas such as the postcentral (primary somatosensory), precentral (primary motor), superior frontal, and superior temporal cortices. Another study observed that resting-state functional connectivity between the lateral portion of the right lobule VI and the right temporoparietal junction was lower in healthy individuals with high worry-proneness than in those with low worry-proneness (Zhang et al., 2024). The right temporoparietal junction is a multimodal association cortex involved in reorienting attention to salient sensory stimuli and detecting violations of expectations (Abu-Akel and Shamay-Tsoory, 2011). Taken together, the abnormal spontaneous activity observed in cerebellar lobule VI of PDFoG patients within the slow-5 and slow-4 bands might reflect the involvement of different subregions of lobule VI in distinct intrinsic brain networks.

Compared to HCs, PDFoG patients showed increased spontaneous activity in several visual brain regions, including the calcarine fissure and surrounding cortex, fusiform gyrus, and cuneus. Specifically, spontaneous activity was increased in the right fusiform gyrus and right cuneus in both the conventional and slow-4 bands. In the bilateral calcarine cortex, increased activity was observed across all three bands in the left hemisphere and in the conventional and slow-5 bands in the right hemisphere. The primary visual cortex (V1), located medially along the calcarine fissure in the occipital lobe, receives input from the retina and is responsible for retinotopic mapping and early visual feature extraction (e.g., edges, color, motion direction, and depth). It then distributes this information *via* the ventral “what” pathway for object recognition and the dorsal “where” pathway for spatial localization (Ng et al., 2006). The cuneus contains both striate (V1) and extrastriate regions (Cohen, 2011) and plays a key role in maintaining stable and coherent visual perception despite constant eye movements—a process known as transsaccadic perception. This function involves tracking object features (shape, orientation) during saccades to aid in object recognition (Baltaretu et al., 2023). The cuneus also acts as a hub linking V1 with extrastriate areas and contributes to depth perception, which is crucial for spatial navigation and interaction with three-dimensional objects (He et al., 2021). The fusiform gyrus, situated on the basal surface of the temporal and occipital lobes, is structurally connected to the cuneus by a white matter tract (Palejwala et al., 2020). It houses the fusiform face area—more prominent in the right hemisphere—which is involved in human face and object recognition as part of the ventral temporal network (Haxby et al., 2001). Hyperactivity in these visual areas may indicate that visual object perception and recognition are disrupted in PDFoG patients. This idea is broadly consistent with a previous study showing that resting-state functional connectivity within the visual network was significantly lower in PDFoG patients than in PDnFoG patients and HCs, suggestive of a breakdown in intrinsic visual processing circuitry (Tessitore et al., 2012).

In PDFoG patients, activity in the conventional and slow-5 bands was decreased in the right superior frontal gyrus (medial orbital) and right rectus gyrus compared to PDnFoG patients, and in the right olfactory cortex compared to HCs. In contrast, in PDnFoG patients, activity in the conventional and slow-4 bands in the right superior frontal gyrus (medial orbital) was reduced compared to HCs. According to the AAL atlas, all three structures lie on the ventral surface of the frontal lobe (Rolls et al., 2015). Located within the orbitofrontal cortex, the secondary olfactory cortex supports higher-order olfactory processing, including the integration of odors with other sensory modalities, such as taste, vision, and touch, to generate flavor (Han et al., 2023). It also contributes to odor recognition memory and the emotional appraisal (pleasantness) of odors (Royet et al., 2001; Waymel et al., 2020). The rectus gyrus has been implicated in olfactory function (Postma et al., 2021). As part of the ventromedial prefrontal cortex, it may also contribute to reward-related decision-making (Rolls, 2023). Notably, the presence of hyposmia (reduced sense of smell) at the time of PD diagnosis has been identified as a significant risk factor for the future development of FoG (Lee et al., 2021). In the present study, we observed that activity in the olfactory cortex and rectus gyrus was reduced in the slow-5 band, but not the slow-4 band, in PDFoG patients, suggesting that the slow-5 band may be more sensitive and relevant for detecting changes in olfaction-related brain areas in these patients. Moreover, PDnFoG patients demonstrated diminished activity in the right superior frontal gyrus (medial orbital), but not the olfactory cortex or rectus gyrus, suggesting that olfactory function is relatively well preserved in these patients. Given these findings, further investigation into the role of olfactory dysfunction in the pathophysiology of PDFoG is warranted.

Overall, PDFoG was associated with overactivity in regions that support visual processing and sensorimotor integration, namely, the bilateral postcentral gyri, cerebellar lobule VI, the calcarine cortex, and the right fusiform gyrus and cuneus. These areas may serve as key components of a sensorimotor network essential for normal gait. Within the context of the perceptual malfunction theory of FoG, two explanations may potentially account for this hyperactivity—visual overload and compensatory upregulation. With visual overload, increased resting activity in visual regions may lead to amplified responses during walking, an activity that introduces dynamic visual and motor challenges, thereby disrupting online motor planning and adaptation. Regarding compensatory upregulation, although action-related visual perception may initially be intact, impaired integration of action-relevant visual information might lead to an increase in activity in visual areas in an attempt to improve object perception and recognition during movement. However, as we did not find significant correlations between regional activity and sensor-based gait behavior, this interpretation remains speculative. Longitudinal studies would be beneficial for understanding the development of PDFoG, as they can reveal how the sensorimotor network changes over time in PD patients. Meanwhile, increased activity in visual, somatosensory, and cerebellar regions may also reflect externally triggered locomotion, wherein stepping is guided by external sensory cues to compensate for impaired automatic movement (Hallett, 2008; Nutt et al., 2011). Additionally, the role of reduced activity in olfactory-related regions in FoG development needs further investigation, especially in light of evidence suggesting that central cholinergic deficits may contribute to both olfactory and gait impairments in PD (Bohnen et al., 2010; Rochester et al., 2012).

Finally, we observed that, compared to HCs, PDnFoG patients displayed decreased activity in the basal ganglia, encompassing the bilateral putamen in the slow-5 band and the right caudate and pallidum in both the slow-5 and slow-4 bands. This aligns with previous research suggesting that decreased intra-basal ganglia functional connectivity in the putamen, caudate, and pallidum may be a characteristic of early PD (within 3 years of diagnosis) and is not associated with generalized neurodegeneration (Rolinski et al., 2015). Moreover, a decrease in ALFF in the bilateral putamen is the most robust finding in PD, as demonstrated by two meta-analyses (Jia et al., 2021; Wang et al., 2018). However, in our study, we did not find reduced activity in the bilateral putamen or other basal ganglia structures in PDFoG patients. This contrasts with previous research showing that ALFF was reduced in the bilateral putamen of PDFoG patients relative to that in HCs (Hu et al., 2020; Mi et al., 2017). This discrepancy may stem from differences in disease duration, as significant differences in putamen activity between PDFoG patients and HCs were present when disease duration was not regressed out as a covariate. Also, neither Mi et al. (2017) nor Hu et al. (2020) included disease duration as a covariate in their comparisons between patients and HCs, unlike our analysis. Nonetheless, impaired putamen function is a hallmark of PD, and further research is needed to explore potential heterogeneity in putamen activity across clinical features and disease stages. Moreover, the right caudate and pallidum in PDnFoG patients showed decreased activity in both the slow-5 and slow-4 bands, with peak reductions occurring at distinct MNI coordinates for each band. This may indicate that specific frequency bands are associated with distinct subregions of the caudate and pallidum, suggestive of the presence of functional heterogeneity within basal ganglia structures.

## 5 Limitations and future research

The study had several limitations. First was the small sample size, which limited the generalizability and robustness of the results. Second, we did not perform a correlation analysis between rs-fMRI data and clinical scale scores owing to differences in the medication state (i.e., OFF *vs*. ON states). Future research should aim to maintain a consistent medication state throughout the experiment. In addition, the motion paradigms used (TUG, NPW, and Turning) involved relatively simple scenarios that may not adequately reflect the complex situations encountered in daily life. Moreover, the duration of these paradigms may have been too short to fully capture patients’ real-world experiences of freezing episodes.

In the current study, PDFoG patients showed more extensive functional alterations in cortical and cerebellar regions than PDnFoG patients. Furthermore, PDFoG was characterized by hyperactivity in sensorimotor areas, including the primary somatosensory cortex, visual processing regions, and cerebellar lobule VI. These findings potentially support the perceptual malfunction theory of FoG. Additionally, the presence of sensorimotor hyperactivity alongside the absence of basal ganglia impairment in PDFoG patients may partly align with the loss of motor automaticity theory of FoG. We also observed reduced activity in olfaction-related regions in PDFoG patients, a finding that warrants further study. Altogether, these results identify the cerebral cortex and cerebellum as key targets for future research into the neural mechanisms underlying PDFoG.

## Data Availability

The original contributions presented in the study are included in the article/Supplementary material, further inquiries can be directed to the corresponding author/s.

## Footnotes

1
http://restfmri.net/forum/restplus
