# Supplementary material for: Gait dynamics and brain function abnormalities in Parkinson’s disease with freezing of gait: a clinical study using resting-state fMRI and wearable devices
Source: Front Neurosci. 2025 Jul 3;19:1560333. doi: 10.3389/fnins.2025.1560333 (PMC12267242; doi:10.3389/fnins.2025.1560333)
Supplement: Supplementary file 1 [file Data_Sheet_1.docx]

Supplementary Material

# Supplementary Figures


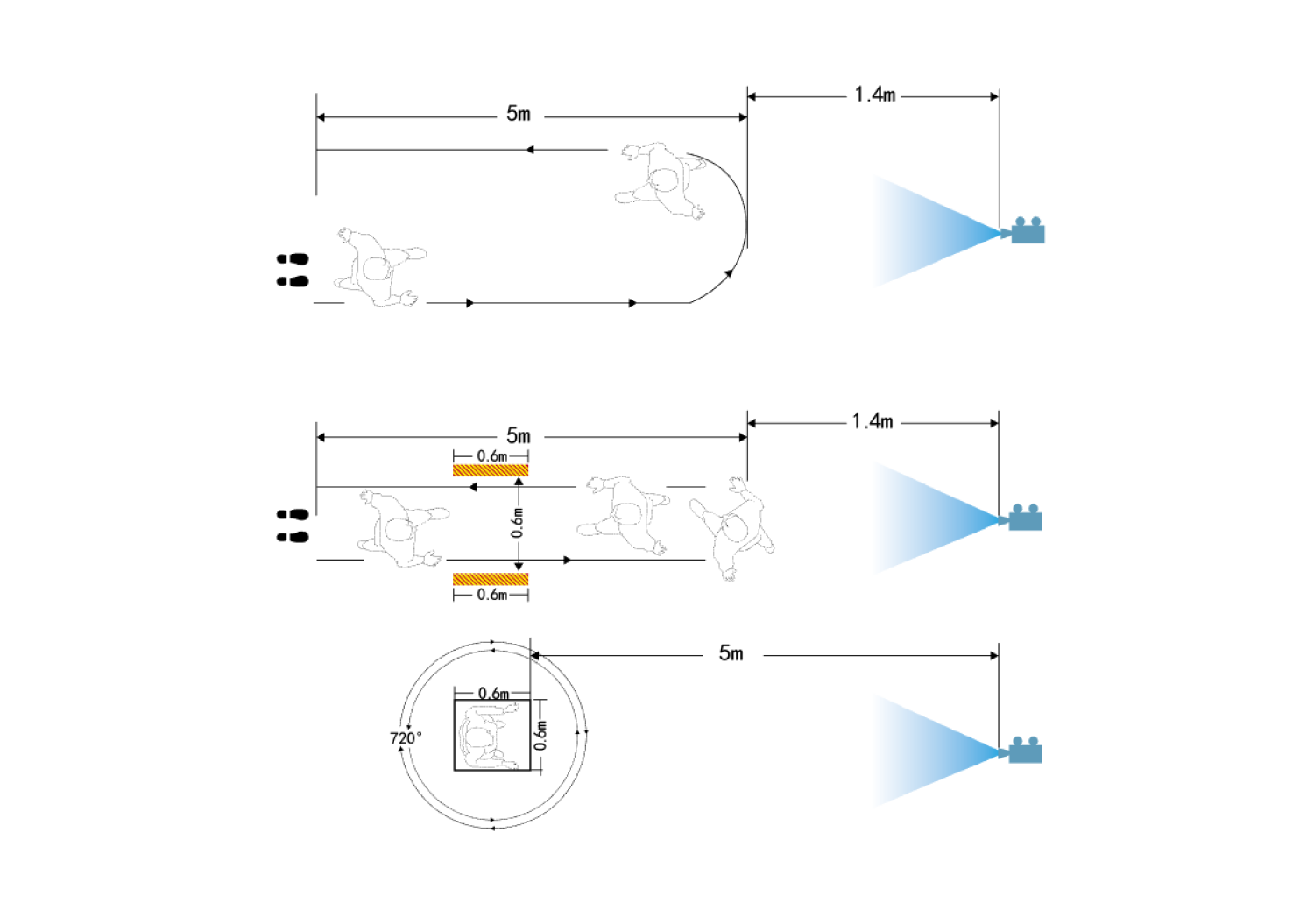


**Supplementary Figure 1.** A picture illustrating the Timed Up and Go (TUG; top), Narrow Path Walking (NPW; middle) and Turning (bottom) gait paradigms.


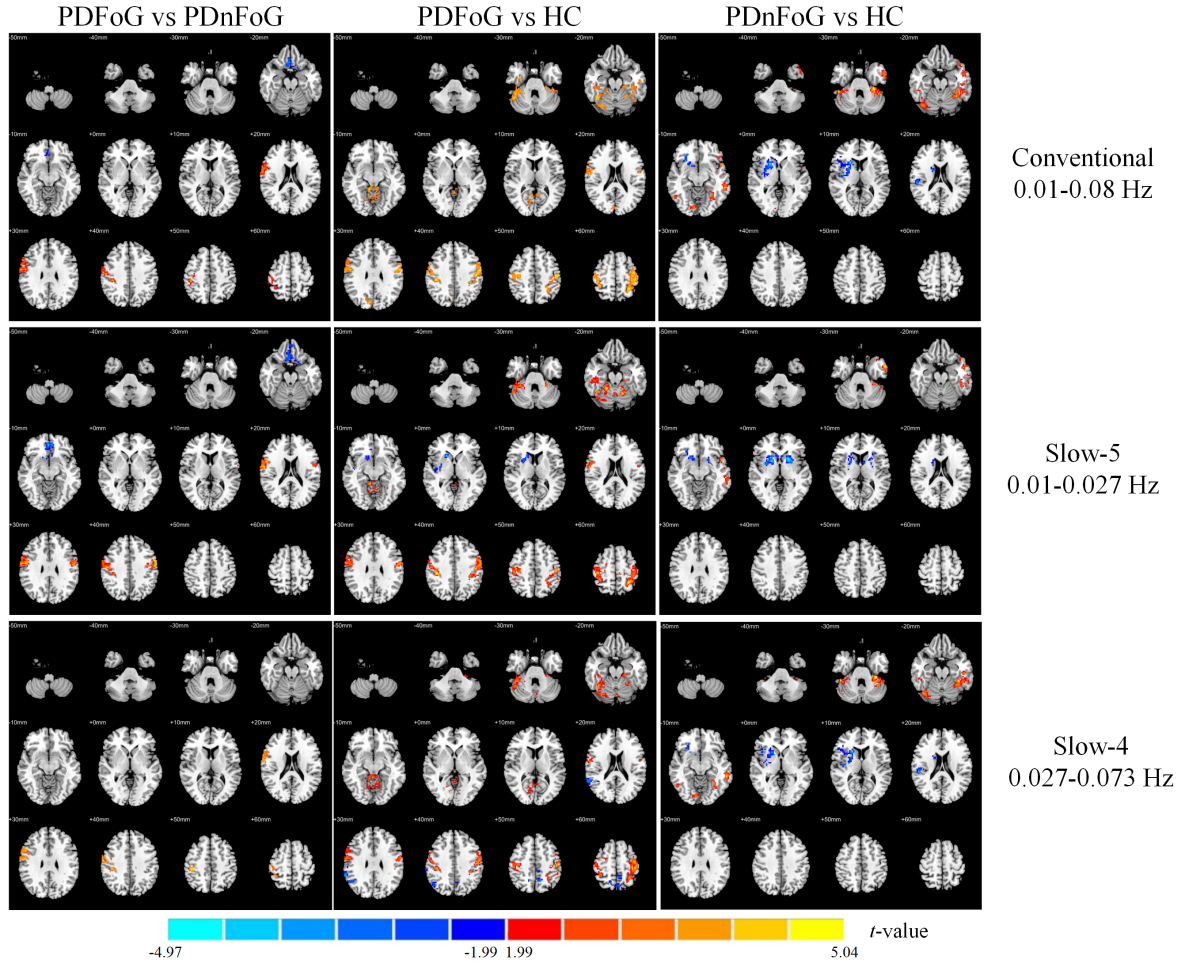


**(A)**


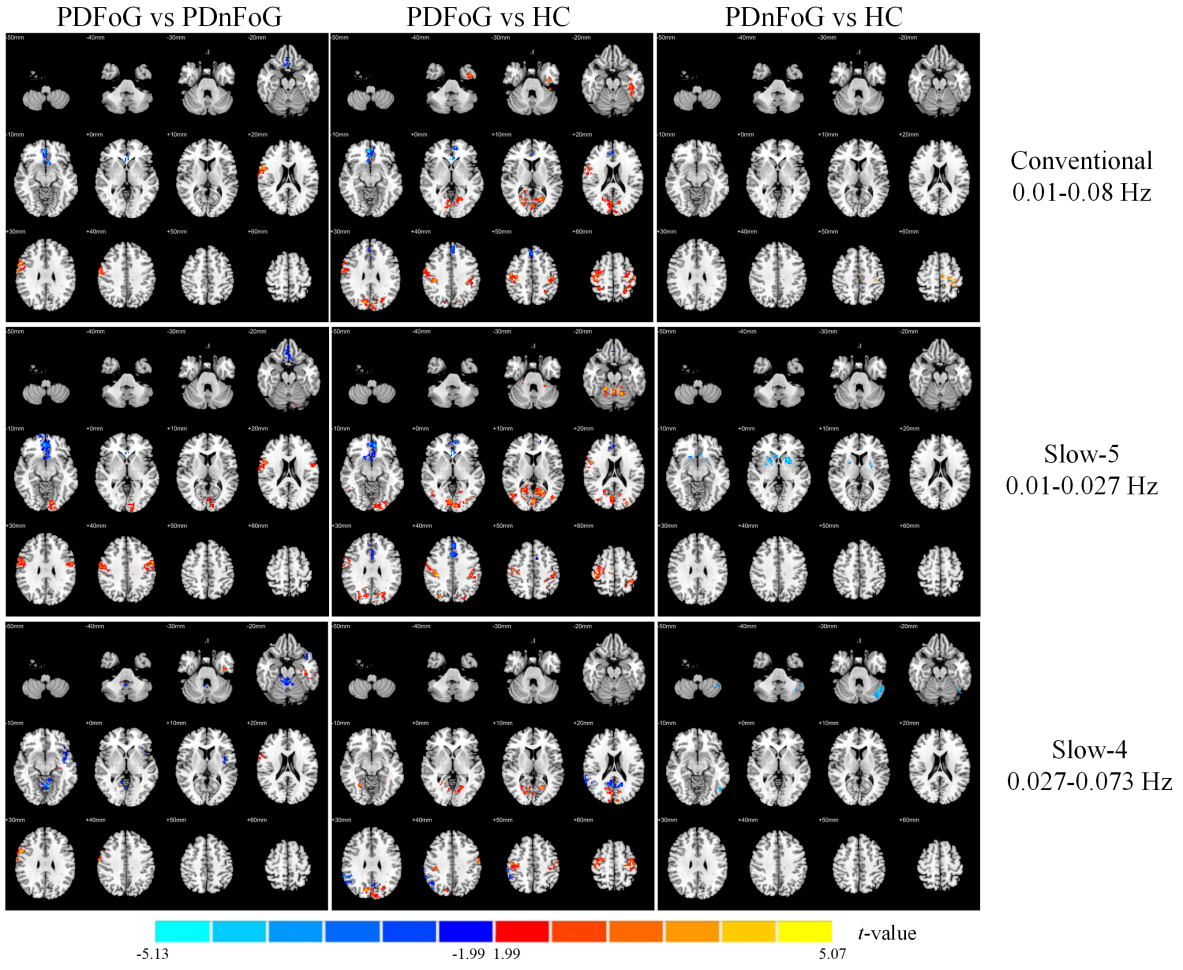


**(B)**


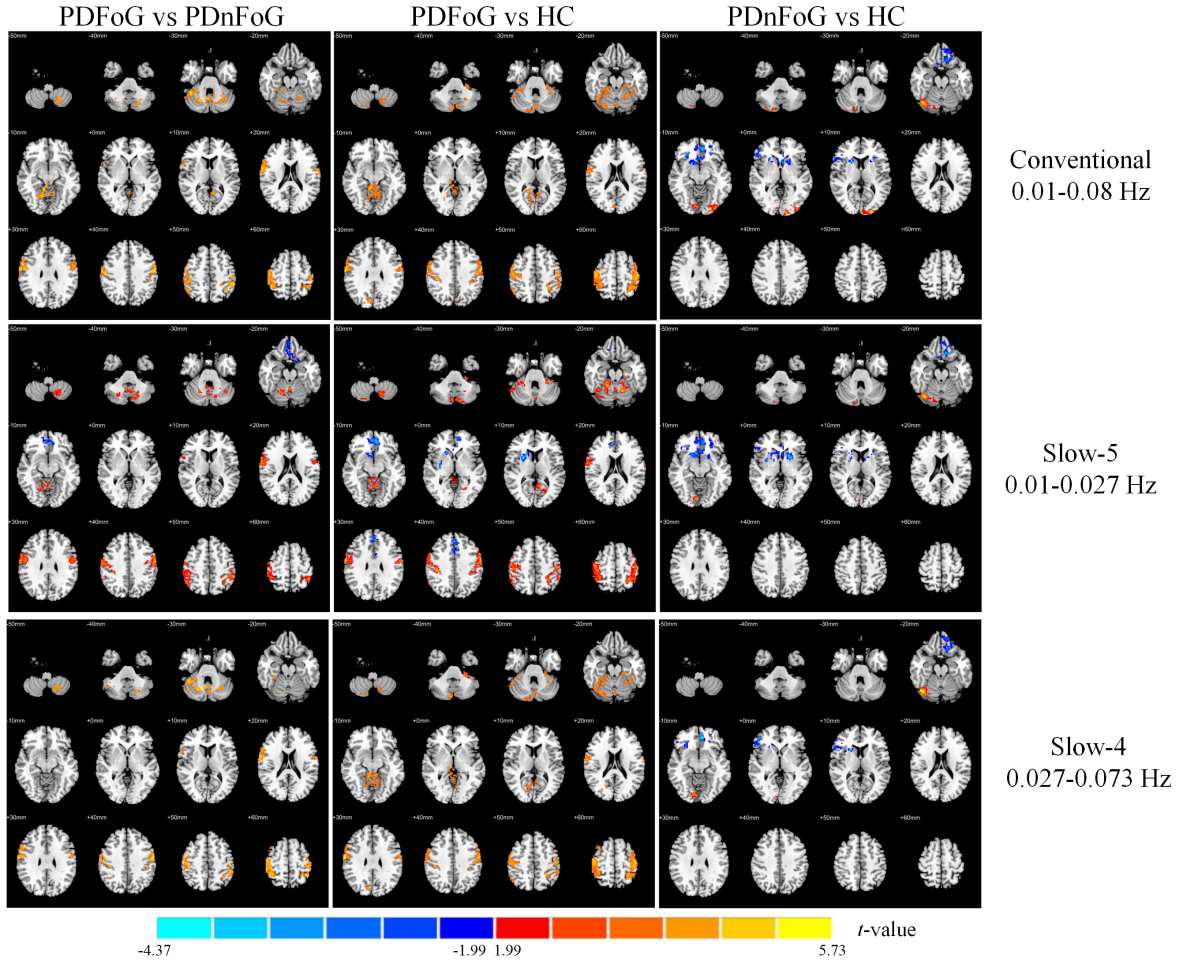


**(C)**


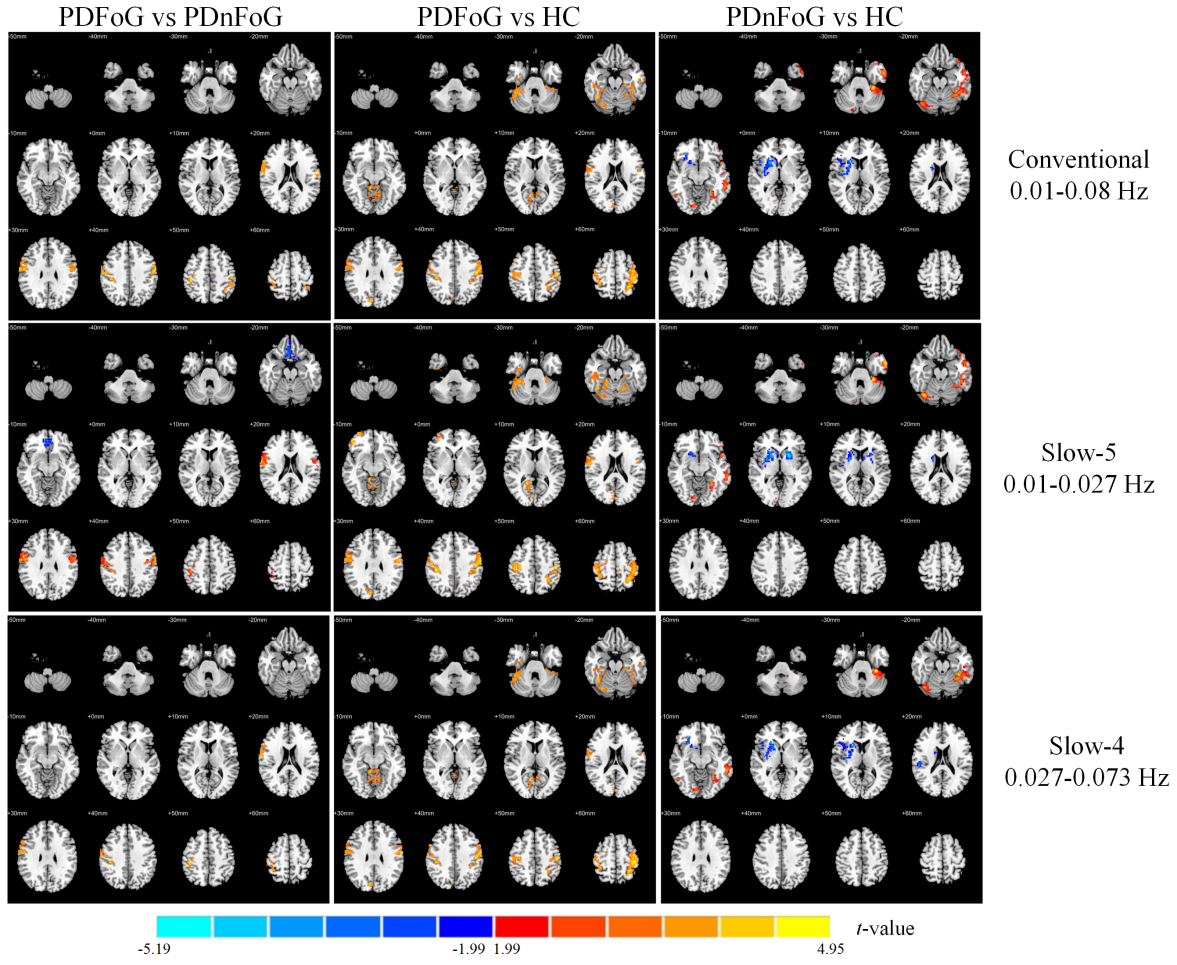


**(D)**

**Supplementary Figure 2.** Group differences in ALFF **(A)**, fALFF **(B)**, PerAF **(C)**, and wavelet-ALFF **(D)** across three frequency bands for PDFoG vs PDnFoG, PDFoG vs HC, and PDnFoG vs HC, after controlling for mean framewise displacement. PDFoG = patients with Parkinson’s Disease and freezing of gait; PDnFoG = patients with Parkinson’s Disease and no freezing of gait; HC = healthy controls.


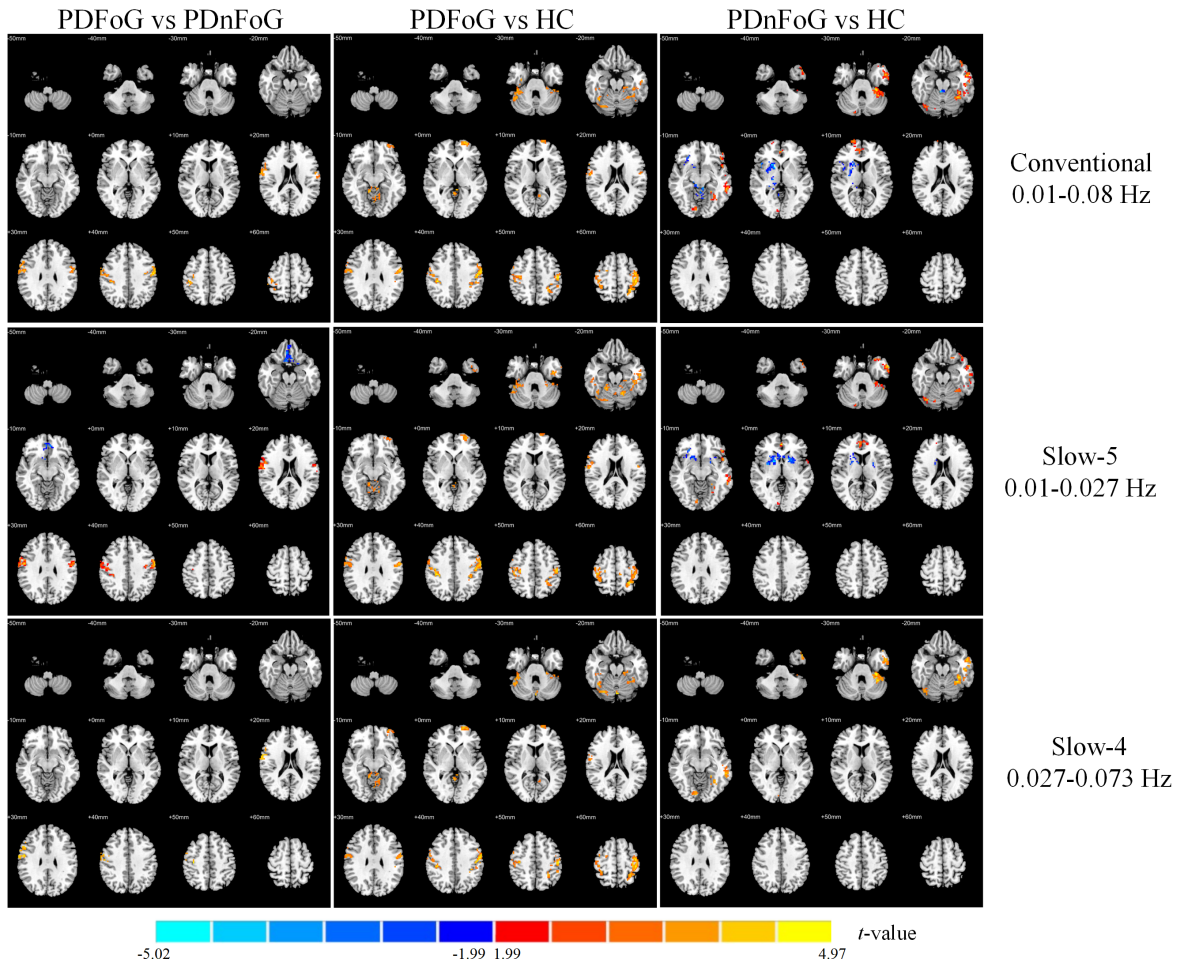


**(A)**


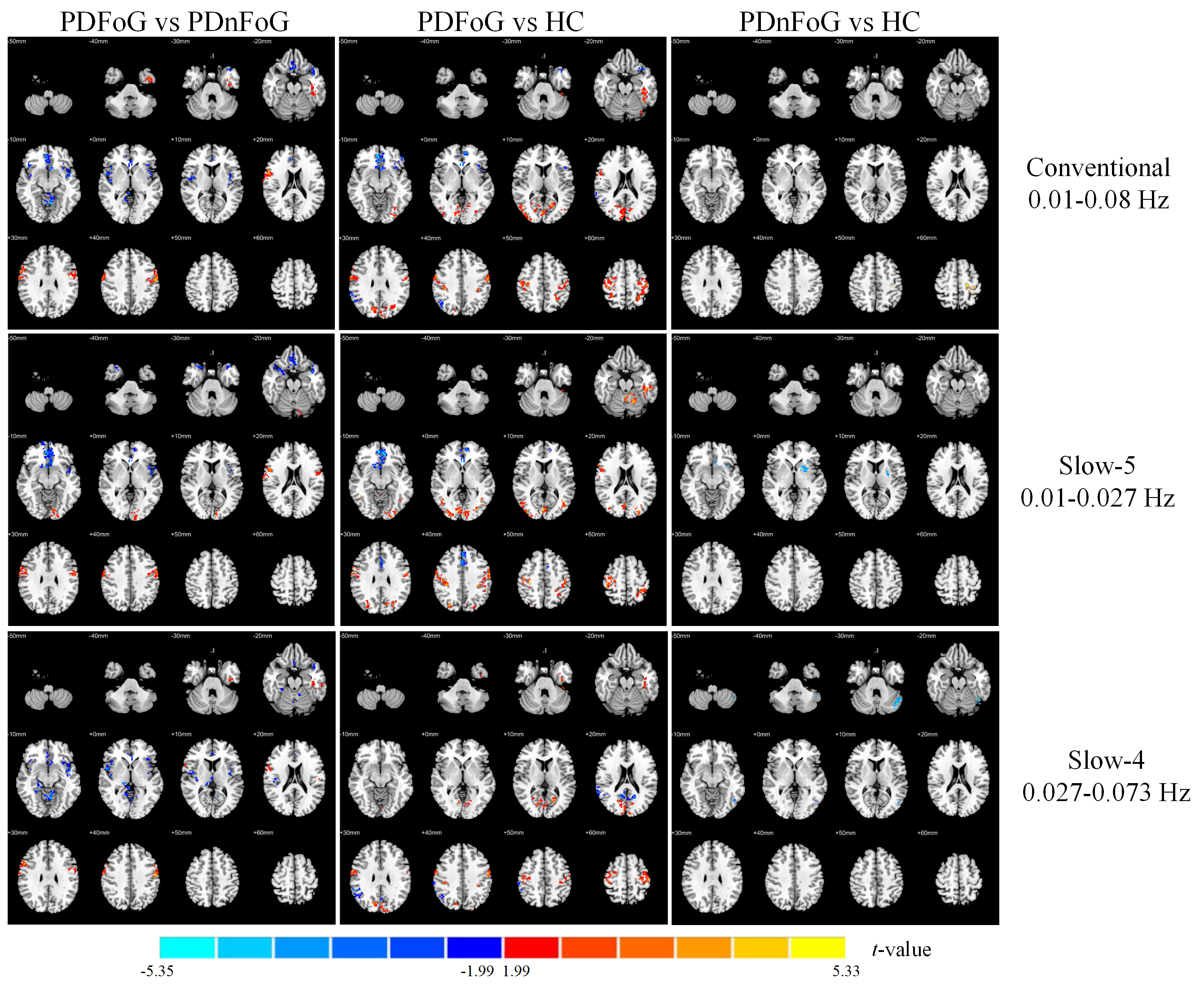


**(B)**


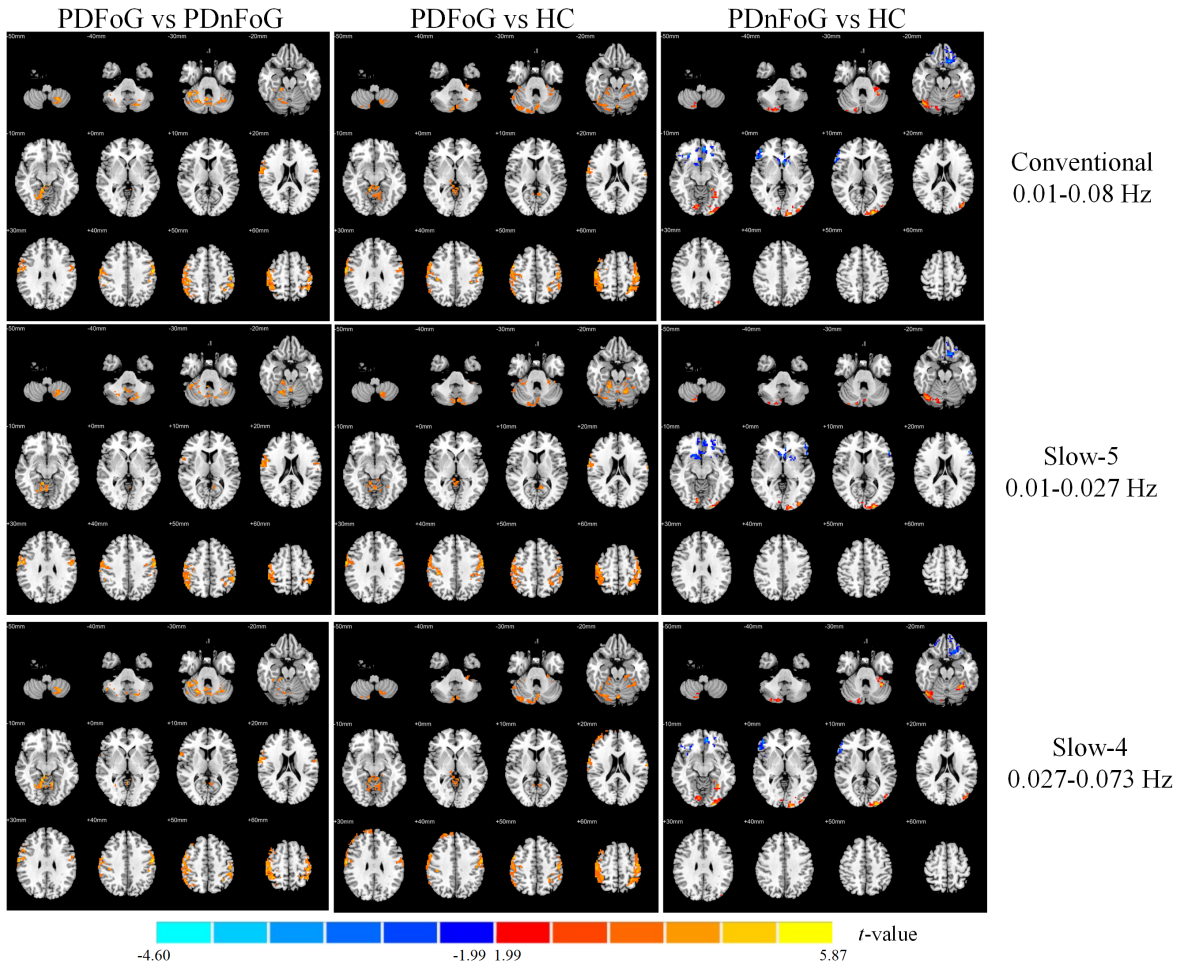


**(C)**


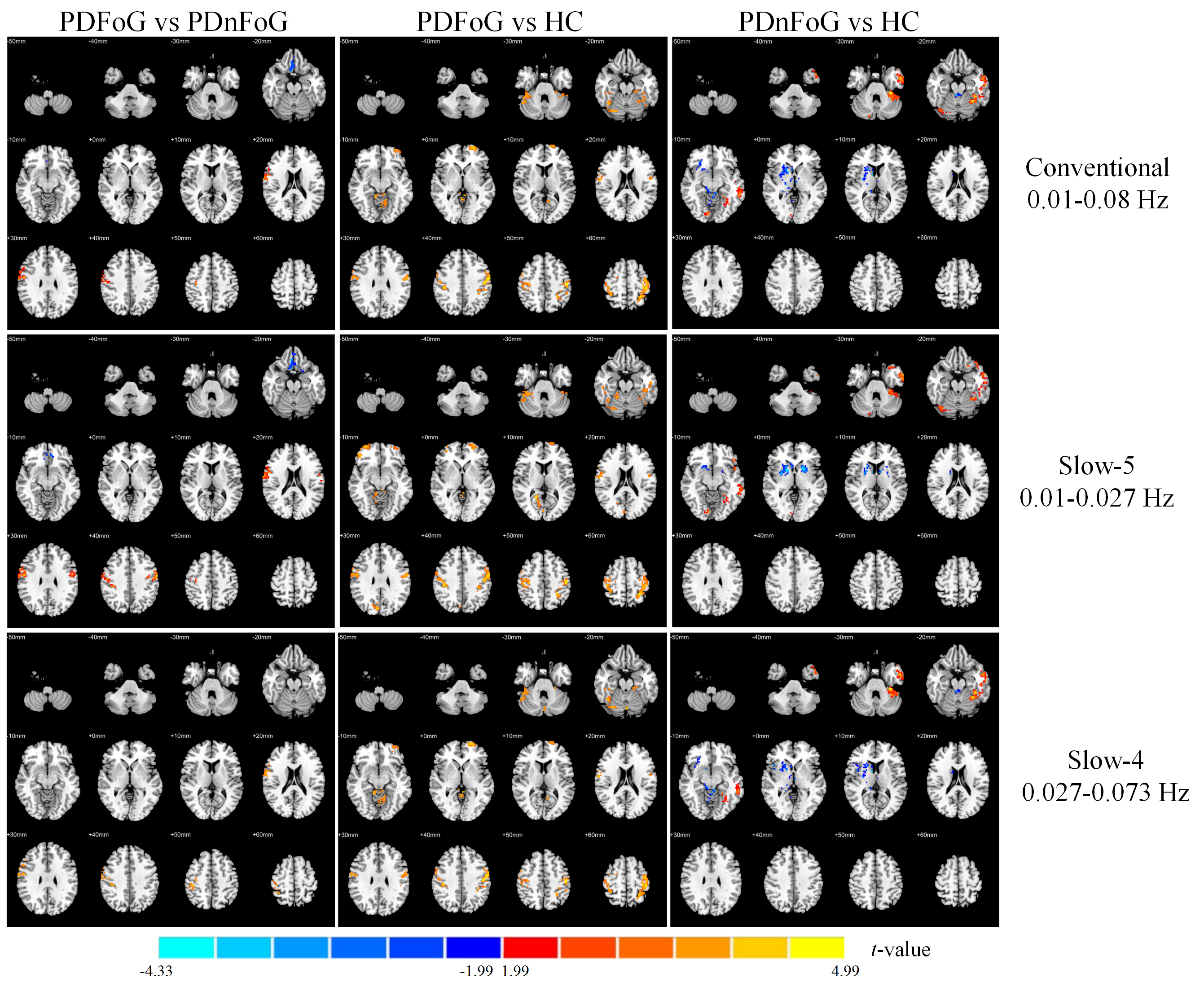


**(D)**

**Supplementary Figure 3.** Group differences in ALFF **(A)**, fALFF **(B)**, PerAF **(C)**, and wavelet-ALFF **(D)** across three frequency bands for PDFoG vs PDnFoG, PDFoG vs HC, and PDnFoG vs HC, using a 4 mm full-width at half-maximum (FWHM) smoothing kernel. PDFoG = patients with Parkinson’s Disease and freezing of gait; PDnFoG = patients with Parkinson’s Disease and no freezing of gait; HC = healthy controls.


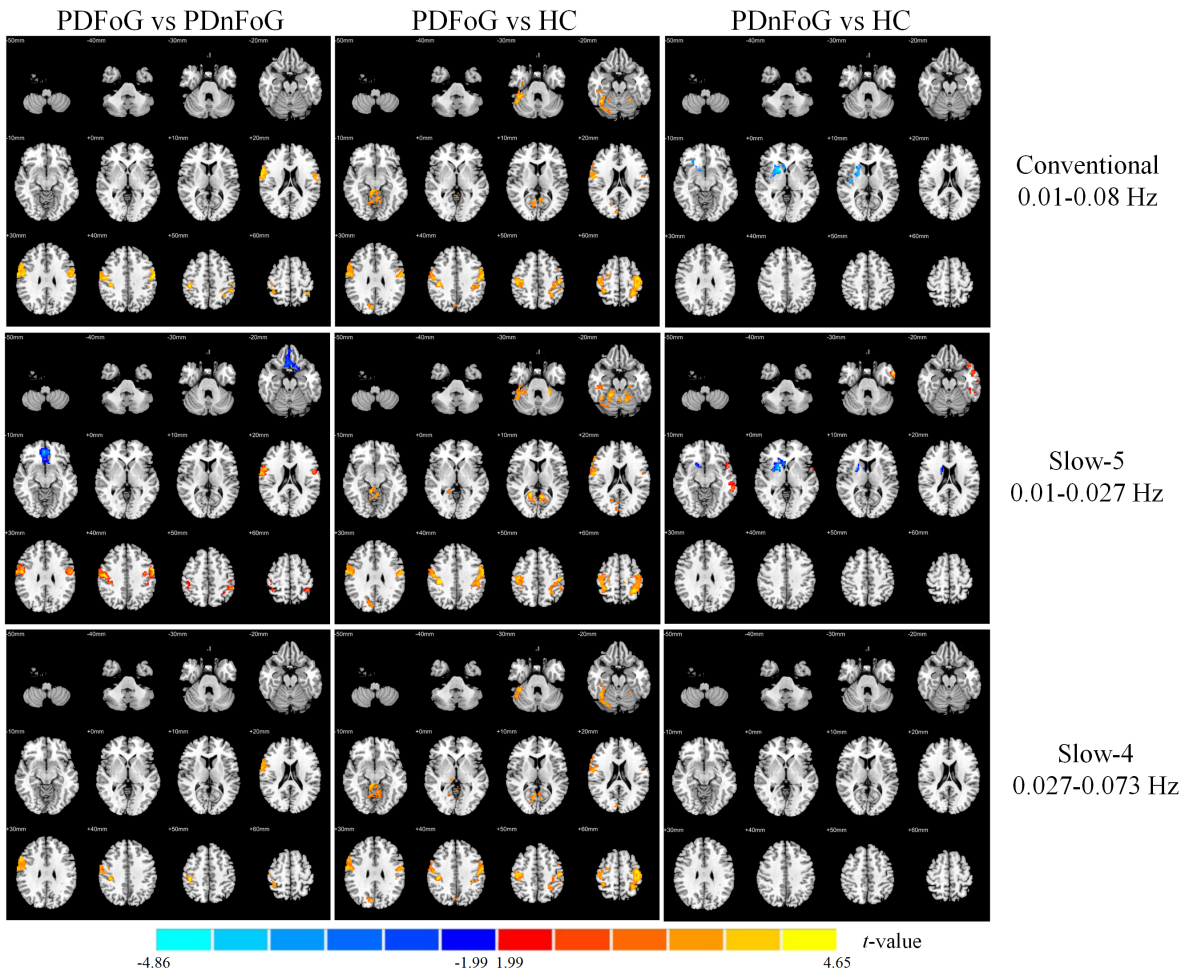


**(A)**


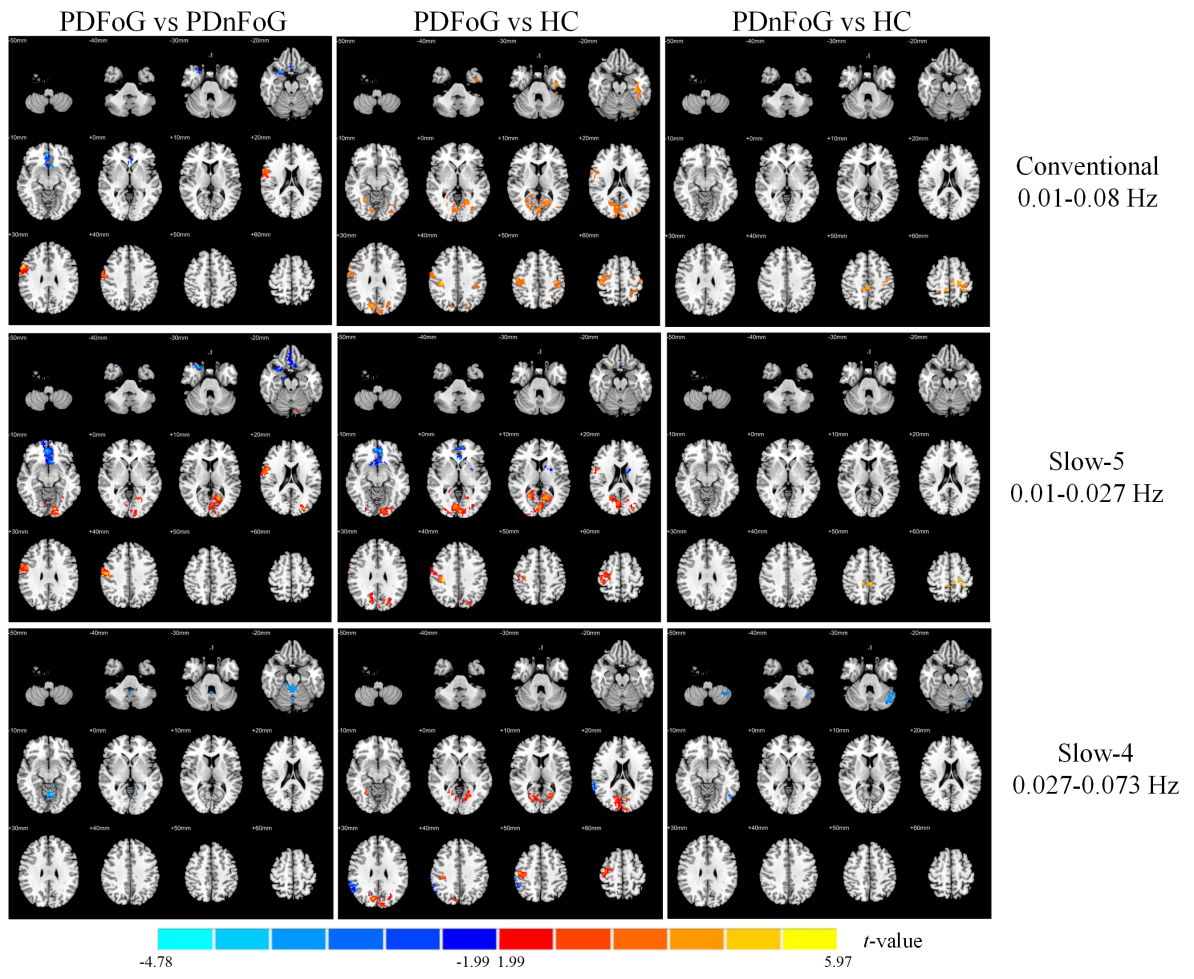


**(B)**


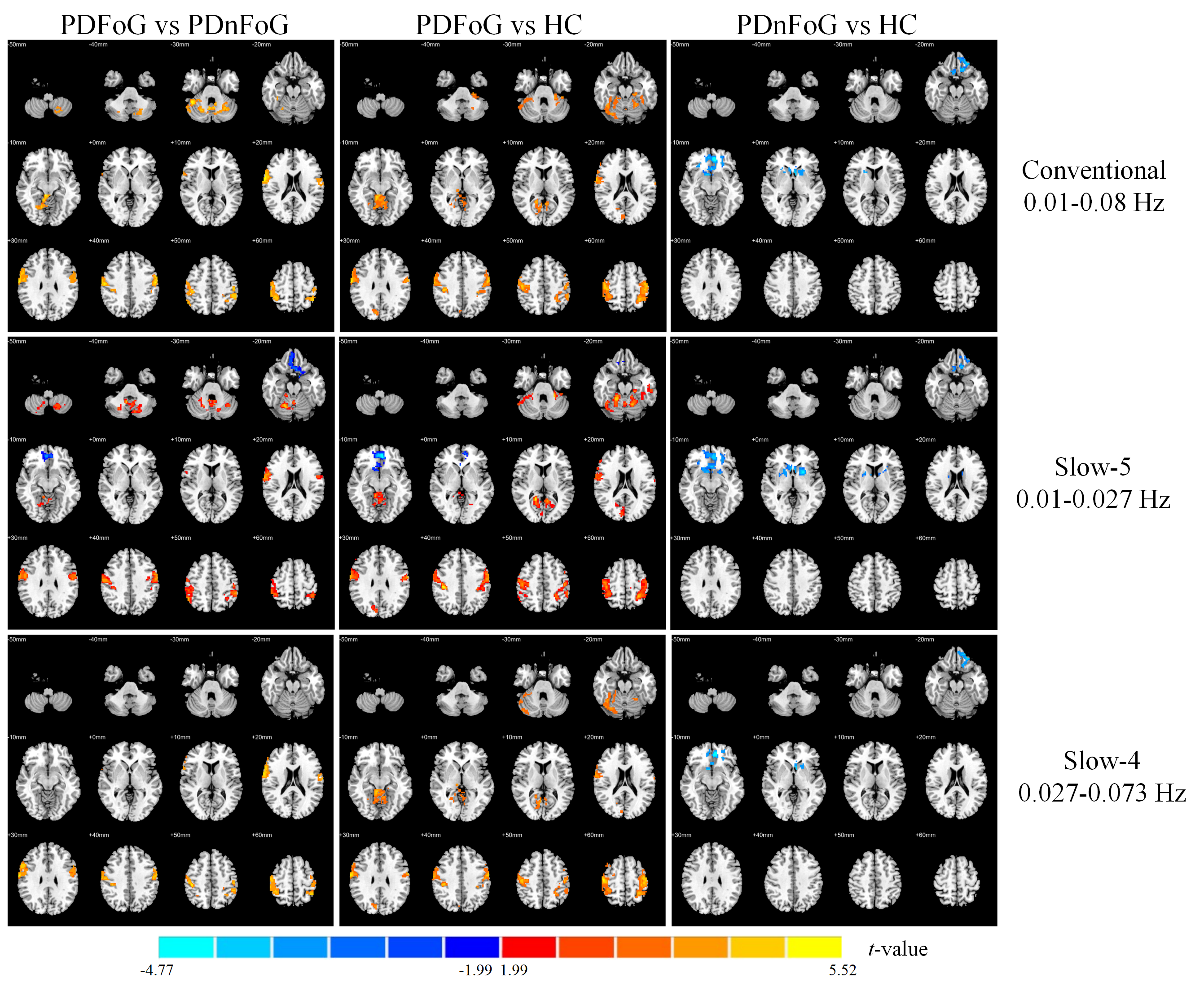


**(C)**


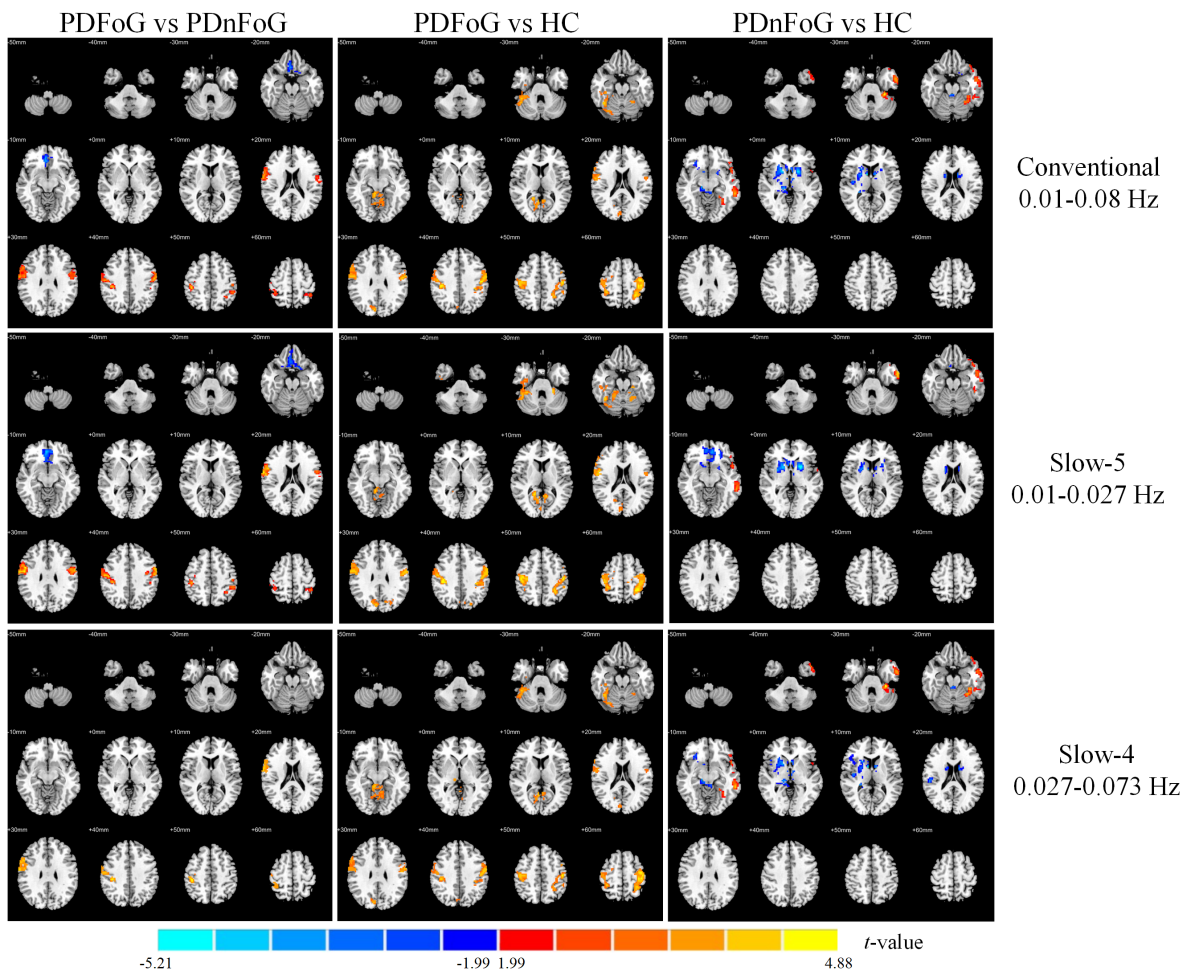


**(D)**

**Supplementary Figure 4.** Group differences in ALFF **(A)**, fALFF **(B)**, PerAF **(C)**, and wavelet-ALFF **(D)** across three frequency bands for PDFoG vs PDnFoG, PDFoG vs HC, and PDnFoG vs HC, using a 8 mm full-width at half-maximum (FWHM) smoothing kernel. PDFoG = patients with Parkinson’s Disease and freezing of gait; PDnFoG = patients with Parkinson’s Disease and no freezing of gait; HC = healthy controls.
